# Functional Genomic Analysis of the *let-7* Regulatory Network in *Caenorhabditis elegans*

**DOI:** 10.1371/journal.pgen.1003353

**Published:** 2013-03-14

**Authors:** Shaun E. Hunter, Emily F. Finnegan, Dimitrios G. Zisoulis, Michael T. Lovci, Katya V. Melnik-Martinez, Gene W. Yeo, Amy E. Pasquinelli

**Affiliations:** 1 Division of Biology, University of California San Diego, La Jolla, California, United States of America; 2 Department of Cellular and Molecular Medicine, University of California San Diego, La Jolla, California, United States of America; 3 Stem Cell Program, University of California San Diego, La Jolla, California, United States of America; 4 Institute for Genomic Medicine, University of California San Diego, La Jolla, California, United States of America; University of California San Francisco, United States of America

## Abstract

The let-7 microRNA (miRNA) regulates cellular differentiation across many animal species. Loss of *let-7* activity causes abnormal development in *Caenorhabditis elegans* and unchecked cellular proliferation in human cells, which contributes to tumorigenesis. These defects are due to improper expression of protein-coding genes normally under *let-7* regulation. While some direct targets of *let-7* have been identified, the genome-wide effect of *let-7* insufficiency in a developing animal has not been fully investigated. Here we report the results of molecular and genetic assays aimed at determining the global network of genes regulated by *let-7* in *C. elegans*. By screening for mis-regulated genes that also contribute to *let-7* mutant phenotypes, we derived a list of physiologically relevant potential targets of *let-7* regulation. Twenty new suppressors of the rupturing vulva or extra seam cell division phenotypes characteristic of *let-7* mutants emerged. Three of these genes, *opt-2, prmt-1*, and T27D12.1, were found to associate with Argonaute in a *let-7*–dependent manner and are likely novel direct targets of this miRNA. Overall, a complex network of genes with various activities is subject to *let-7* regulation to coordinate developmental timing across tissues during worm development.

## Author Summary

In the past decade, microRNAs (miRNAs) have become recognized as key regulators of gene expression in many biological pathways. These small, non-coding RNAs target specific protein-coding genes for repression. The specificity is mediated by partial base-pairing interactions between the 22 nucleotide miRNA and sequences in the target messenger RNA (mRNA). The use of imperfect base-pairing means that a single miRNA can regulate many different mRNAs, but it also means that identifying these targets is not straightforward. One of the first discovered miRNAs, let-7, generally promotes cellular differentiation pathways through a repertoire of targets that is yet to be fully described. Here we utilized molecular and genetic approaches to identify biologically relevant targets of the let-7 miRNA in *Caenorhabditis elegans*. Our analyses indicate that let-7 regulates a large cast of genes, both directly and indirectly. Loss of *let-7* activity in *C. elegans* results in multiple developmental abnormalities and, ultimately, death. We uncovered new targets of let-7 that contribute to these phenotypes when they fail to be properly regulated. Given the highly conserved nature of let-7 from worms to humans, our studies highlight new genes and pathways potentially under let-7 regulation across species.

## Introduction

MicroRNAs (miRNAs) are an abundant class of regulatory genes that control many cellular and developmental processes [1]. The biogenesis of miRNAs requires multiple steps, beginning with transcription by RNA polymerase II to produce capped and polyadenylated primary transcripts [2], [3]. These transcripts are processed sequentially by the RNase III enzymes Drosha and Dicer, resulting in the ∼22 nucleotide (nt) single stranded mature miRNA. The mature miRNA is incorporated into the RNA induced silencing complex (RISC), which uses the miRNA as a sequence specific guide to find and mediate regulation of target mRNAs. The miRISC usually induces translational repression and destabilization of the target mRNA through mechanisms that are still being determined [4], [5].

let-7 was originally discovered as a miRNA controlling developmental timing in *Caenorhabditis elegans* [6], [7]. The lethality associated with mutations in this gene is at least partly due to vulval rupturing, where internal organs burst out of the egg-laying pore. Additionally, lateral hypodermal seam cells fail to terminally differentiate at the larval to adult transition in *let-7* mutants. These phenotypes place *let-7* in the heterochronic pathway, which includes genes that regulate the temporal identity of cell divisions and fates [6], [8]. *let-7* regulates developmental timing, in part, through the direct target genes *lin-41* and *hbl-1* [6], [7], [9], [10]. These genes, in turn, regulate the transcription factor *lin-29*, which directly controls terminal differentiation in the hypodermis [6], [7], [9], [10]. Several transcription factors, such as the nuclear hormone receptor *daf-12*, the forkhead transcription factor *pha-4* and the zinc finger protein *die-1*, genetically interact with *let-7* and are also likely direct targets [11]. Genetic mutation or RNAi depletion of any one of these *let-7* targets is sufficient to at least partially rescue the lethality of *let-7* mutants.

The let-7 miRNA is a widely conserved animal miRNA and its role in regulating differentiation also appears to be conserved [12], [13], [14]. Typically, expression of let-7 family miRNAs is negligible in stem cells and in early embryonic tissues and is then up-regulated as cells take on more differentiated fates. In worms and mammalian cells, the LIN-28 RNA binding protein is largely responsible for keeping let-7 miRNA levels low during early development [15]. LIN-28 prevents the maturation of let-7 family miRNAs by blocking Drosha or Dicer processing or promoting destabilization of let-7 precursors [16], [17], [18], [19], [20], [21], [22], [23]. The abnormally low expression of let-7 detected in various types of tumors has been linked, in some cases, to aberrant up-regulation of LIN-28 [24]. Additionally, let-7 and LIN-28 have opposing effects on insulin sensitivity in mice [25], [26]. This is due at least in part to direct targeting of several metabolic genes by let-7 miRNA.

Consistent with its role in promoting differentiated states, decreased expression of let-7 miRNA has been associated with numerous types of cancer [14]. In fact, one of the first discovered targets of let-7 in humans is *RAS*, a notorious oncogene [27]. Since then, many genes that promote cell division or antagonize the differentiated state have been implicated as direct or indirect targets of let-7 regulation [28], [29], [30], [31], [32], [33]. Remarkably, the introduction of let-7 miRNA into lung or breast tumors in mouse models has been shown to halt tumor growth *in vivo* [31], [34], [35]. Thus, let-7 functions as a tumor suppressor in at least in some settings, where it represses the expression of genes needed for oncogenesis.

To understand how let-7 or any miRNA controls a cellular process, the genes it regulates must be identified. Many computational prediction approaches have been taken to match miRNAs to targets [11], [36], [37], [38], [39], [40], [41]. However, the limited overlap of predicted targets between programs suggests that a consensus regarding the rules for target recognition is yet to be reached. The best defined motif for target recognition is perfect pairing of miRNA nucleotides 2–7, called the “seed” region, with a target sequence [42]. Deviations from seed pairing can be compensated for by strong pairing of the 3′ end of the miRNA or “centered sites”, where the middle portion of the miRNA forms consecutive base pairs with the target [42], [43]. Several validated target sites fail to conform to any of these motifs [42], [44]. Furthermore, other features, such as location within an mRNA and RNA secondary structure surrounding the miRNA complementary sequence influence whether a target site will be recognized *in vivo*. Ultimately, the endogenous context of the target site and the cellular environment will determine which sequences will be recognized and regulated by miRISC.

Numerous experimental methods have complemented the *in silico* endeavors to match miRNAs with direct targets. Traditional genetic as well as RNAi-based suppressor screens have uncovered major targets of the first described miRNAs in *C. elegans* [6], [7], [11], [27], [45], [46]. More high-throughput methods have been based on the general role of miRNAs in down-regulating mRNA and protein levels of their targets [5], [47]. Microarray or RNA-seq analysis of transcripts up-regulated when a miRNA is absent can provide lists of potential direct targets [48], [49], [50], [51]. Likewise, large-scale proteomics analyses have been used to detect proteins sensitive to changes in expression of specific miRNAs [52], [53], [54]. More recently, ribosome profiling has been developed as an indirect method for assessing changes in the translation status of mRNAs, leading to the conclusion that regulation by miRISC generally results in target mRNA destabilization [55], [56]. A more direct approach for detecting targets of miRISC is to capture mRNAs associated with Argonaute complexes. RNA immunoprecipitation (RIP) or cross-linking followed by IP (CLIP) protocols have been used to identify entire transcripts or the actual mRNA sequences in contact with Argonaute, respectively [57], [58], [59], [60], [61], [62], [63], [64], [65], [66]. These types of experiments demonstrate that an mRNA is recognized by miRISC but do not necessarily reveal the identity of the miRNA involved or if the interaction is regulatory.

We combined several molecular and genetic methods to identify physiologically relevant targets of *let-7* in *C. elegans*. Our approach for discovering new *let-7* regulatory targets takes advantage of *let-7* dependent expression differences of the known targets, including *lin-41* [67], [68]. We postulated that other direct targets would also be mis-regulated in *let-7* mutants. Therefore, *in vivo* expression changes were analyzed in wild-type (WT) and *let-7* mutant animals using microarray analysis to identify a list of relevant candidate target genes. This list of genes was further refined by computational target predictions and expression analysis in the downstream heterochronic mutant, *lin-29*. The relevance of the up-regulated genes for *let-7* phenotypes was tested through RNAi-based suppressor screens. These genetic analyses revealed twenty new downstream effectors of *let-7* phenotypes, including multiple transcription factors and metabolic proteins. Several of these genes also affect *let-7* dependent phenotypes seen in *lin-28* mutants revealing a complex genetic interaction with *let-7*. By showing *let-7* dependent association with Argonaute, we were able to confirm three new direct targets of *let-7* with binding sites in the 3′ UTRs as well as in coding regions.

## Results

**Figure 1:**
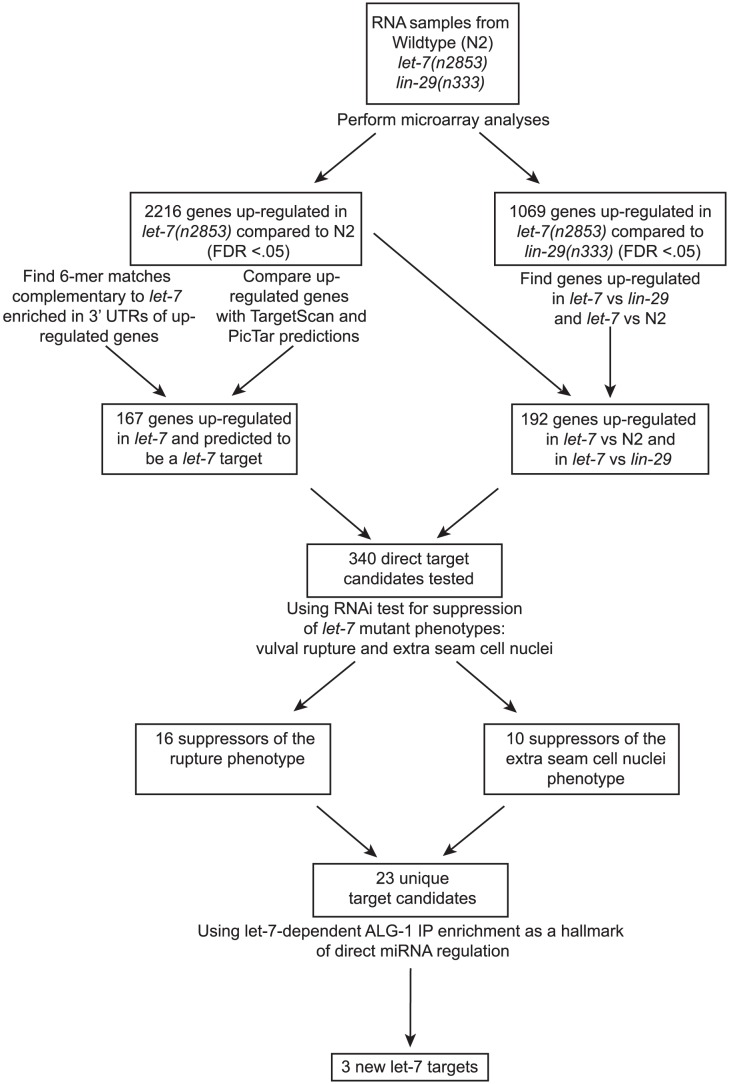
Summary of experimental design and results. Shown is a flowchart outlining the experiments and analyses leading to the discovery of 3 new potential *let-7* targets.

While some direct targets of the *let-7* miRNA are known, a full picture of the *let-7* regulatory network remains largely uncharacterized. Although several groups have attempted to identify *let-7* targets in *C. elegans*, the criteria and, consequently, the predicted targets from these approaches have minimal overlap [11], [36], [38], [39], [40], [41], [69]. We have undertaken a multi-step approach for the discovery and validation of *let-7* targets in *C. elegans*, beginning with analysis of global, *let-7*-dependent gene expression changes, and followed by genetic interaction analysis of candidates. Final validation of direct targets was confirmed by *let-7* dependent RISC association (Figure 1).

### Widespread gene mis-regulation in worms deficient for *let-7* activity

We have previously shown *let-7*-dependent mRNA destabilization of known direct targets [67], suggesting that in addition to giving a general picture of *let-7* function, microarray analysis of gene-misregulation in *let-7* mutants will provide a basis for the discovery of new direct targets. The *let-7(n2853)* mutation changes the fifth G to an A in the mature let-7 miRNA [6], which destabilizes target interactions and results in up-regulation of *lin-41* mRNA, an established *let-7* target [7], [67], [70]. To identify globally the genes regulated by *let-7*, six independent and paired wild-type and *let-7(n2853)* fourth larval stage (L4) RNA samples were labeled and hybridized to Affymetrix arrays. 2216 genes were up-regulated, and 1905 genes were down-regulated in the *let-7(n2853)* mutants compared to WT worms (FDR<0.05) (Table S1). By microarray analysis, most of the differentially expressed genes were only modestly mis-regulated, as only 42 genes were up-regulated >2-fold (Table 1) and 49 were down-regulated by >2-fold (Table 2). Illustrating the role of *let-7* as a master regulator of development, the up-regulated genes were enriched for Biological Process Gene Ontology (GO) terms representing larval growth and development (Table S1). The up-regulated genes represent direct, including the known targets *lin-41, daf-12*, and *hbl-1*, and indirect targets of *let-7* repression. To further investigate the regulatory relationships between *let-7* and the up-regulated genes, a combination of computational and molecular-genetic criteria were used to enrich for direct target candidates among the up-regulated genes.

**Table 1:** Genes up-regulated more than 2-fold in *let-7(n2853)* compared to wild-type.

Gene	Sequence Name^1^	Fold Change	p-value^2^	Target Prediction^3^	Gene Description
*col-90*	C29E4.1	6.06	0.0009		Collagen
*oac-29*	F41E6.14	5.76	0.0004		Integral membrane O-acyltransferase
*col-41*	T10B10.1	3.84	0.0014		Collagen
	F15B9.8	3.1	0.0004		Thrombospondin type 1 domain
	C42D4.3	3.02	0.0067		Fibronectin
	Y47D7A.13	2.91	0.0084		Unknown function
*pqn-5*	C03A7.4	2.88	0.0129		Prion-like-(Q/N-rich)-domain-bearing protein
*dct-5*	F07F6.5	2.86	0.0130		DAF-16/FOXO Controlled, germline Tumor affecting
*lin-41*	C12C8.3	2.8	0.0001	W RGMC	Ring finger-B box-Coiled coil
	K01D12.9	2.8	0.0161		Unknown function
	ZK180.5	2.77	0.0077	W R M	Unknown function
	ZK970.7	2.75	0.0164		DUF148
*abu-6*	C03A7.7	2.73	0.0098		Activated in Blocked Unfolded protein response
	F35B3.4	2.67	0.0153	M	Fibronectin
*grl-21*	ZC168.5	2.55	0.0125		Hedgehog-like protein
*nspb-10*	C01G12.6	2.47	0.0193		Nematode Specific Peptide family, group B
	T20D4.12	2.44	0.0002		DUF19
	C09H5.2	2.41	0.0029	W	Na+/K+ ATPase, alpha subunit
*grd-6*	T18H9.1	2.39	0.0163	W	Hedgehog-like protein
*abu-8*	C03A7.14	2.34	0.0161		Activated in Blocked Unfolded protein response
*grl-4*	F42C5.7	2.34	0.0181		Hedgehog-like protein
*col-91*	F09G8.6	2.27	0.0123	P	Collagen
*clec-52*	B0218.8	2.26	0.0002		C-type lectin
*pgp-14*	F22E10.3	2.26	0.0085	W	Multidrug/pheromone exporter, ABC superfamily
*rnh-1.3*	C04F12.9	2.25	0.0001	W Y	Ribonuclease H
	W08E12.2	2.23	0.0173		Unknown function
*fmo-4*	F53F4.5	2.22	0.0013		Flavin-containing monooxygenase
	C28H8.5	2.19	0.0015	Y	DUF1794
*nspb-12*	F09F7.8	2.18	0.0118	W	Nematode Specific Peptide family, group B
	F10D11.6	2.17	0.0264		BPI/LBP/CETP family protein
*grd-14*	T01B10.2	2.16	0.0227		Hedgehog-like protein
*col-156*	F57B7.3	2.14	0.0050		Collagen
*tyr-2*	K08E3.1	2.14	0.0145		Tyrosinase
	R12E2.7	2.11	0.0476		Unknown function
	W08E12.3	2.11	0.0247		Unknown function
	C29F3.3	2.11	0.0194		Unknown function
*abu-10*	F35A5.3	2.11	0.0195		Activated in Blocked Unfolded protein response
	F18E9.3	2.1	0.0115		Unknown function
*daf-12*	F11A1.3	2.1	0.0003	WPYTRGMC	Nuclear hormone receptor
*col-54*	F33D11.3	2.05	0.0074	W	Collagen
	T28C12.4	2.05	0.0151	M	Carboxylesterase and related proteins
	K07E1.1	2.03	0.0271	P	ARD/ARD′ family

1 Sequence names from WormBase (http://www.wormbase.org).

2 FDR corrected.

3 W = mirWIP [41], P = PITA [40], Y = (this study), T = TargetScan [69], R = RNA22 [39], G = MicroTarget [11], M = Miranda [36], C = PicTar [38].

**Table 2:** Genes down-regulated more than 2-fold in *let-7(n2853)* compared to wild-type.

Gene	Sequence Name^1^	Fold Change	p-value^2^	Target Prediction^3^	Gene Description
*col-38*	F54C9.4	17.47	0.0018		Collagen
*bli-1*	C09G5.6	11.84	0.0035		Collagen
*col-175*	C35B8.1	11.8	0.0060		Collagen
*bli-1*	C09G5.6	11.41	0.0035		Collagen
	E01G4.6	10.81	0.0096		Unknown function
*col-49*	K09H9.3	9.39	0.0099		Collagen
*col-71*	Y49F6B.10	7.23	0.0060		Collagen
*sta-2*	F58E6.1	7.16	0.0041		STAT transcription factor family
*rol-1*	Y57A10A.11	6.07	0.0093		Collagen
	C33C12.3	5.73	0.0000		Beta-glucocerebrosidase family
*dao-4*	ZC373.6	5.44	0.0140	W	Dauer or Aging adult Overexpression family
*col-138*	C52D10.13	4.87	0.0067	W	Collagen
*bli-2*	F59E12.12	4.56	0.0184		Collagen
	T06D8.1	4.01	0.0008	W	Unknown function
*vit-6*	K07H8.6	3.91	0.0002	W M	Vitellogenin
	D1014.7	3.9	0.0095		Unknown function
	D1014.6	3.32	0.0130		Unknown function
	B0393.5	3.15	0.0211		Unknown function
*col-109*	Y38C1BA.3	3.13	0.0257		Collagen
	Y71G12B.18	3.01	0.0113		Unknown function
	R01E6.5	2.96	0.0410		Unknown function
	F09C8.1	2.95	0.0021		Phospholipase
*vit-1*	K09F5.2	2.88	0.0008		Vitellogenin
*col-48*	Y54E10BL.2	2.84	0.0276		Collagen
	Y39B6A.9	2.76	0.0180		Unknown function
	B0222.10	2.71	0.0232		Unknown function
*col-63*	ZK265.2	2.7	0.0107		Collagen
*col-109*	Y38C1BA.3	2.53	0.0257		Collagen
*mltn-12*	C53B4.8	2.51	0.0161	P	MLt-TeN (mlt-10) related
*col-104*	F58F6.1	2.45	0.0402		Collagen
	Y40H7A.10	2.42	0.0005		Cysteine proteinase Cathepsin L family
*col-110*	F19C7.7	2.41	0.0142		Collagen
*vit-2*	C42D8.2	2.37	0.0030		Vitellogenin
	ZK105.1	2.34	0.0014		Unknown function
*cut-3*	F22B5.3	2.32	0.0494		Cuticulin
	F19H6.5	2.28	0.0252		Unknown function
*ugt-47*	R04B5.9	2.28	3E-06		UDP-glucuronosyltransferase family
	ZK512.7	2.26	0.0002		Unknown function
	D1086.3	2.24	0.0006		Unknown function
*col-97*	ZK1010.7	2.22	0.0338	W	Collagen
	F55C10.4	2.2	0.0199		Unknown function
	F31D5.2	2.19	0.0033		Unknown function
*mab-3*	Y53C12B.5	2.19	0.0063		Transcription Factor
*col-79*	C09G5.3	2.17	0.0392		Collagen
	F01G10.9	2.14	0.0303		Unknown function
	K02B12.6	2.11	0.0301		Unknown function
*nhr-234*	Y38E10A.18	2.09	0.0019		Nuclear hormone receptor
	Y46G5A.29	2.05	0.0443		Unknown function
	W03D2.9	2.02	0.0138		Unknown function

1 Sequence names from WormBase (http://www.wormbase.org).

2 FDR corrected.

3 W = mirWIP [41], P = PITA [40], Y = (this study), T = TargetScan [69], R = RNA22 [39], G = MicroTarget [11], M = Miranda [36], C = PicTar [38].

### Enrichment of *let-7* complementary sequences in the 3′ UTRs of genes up-regulated in *let-7* mutants

Direct mRNA targets of miRNAs typically have partially complementary miRNA binding sites, making prediction of miRNA targets from genomic sequence difficult [42], and many groups have developed a variety of rules for target recognition [11], [36], [38], [39], [40], [41], [69]. To enrich for biologically relevant candidates and allow for non-canonical binding sites, we searched for enriched 6-mer sequences in the 3′ UTRs of the genes up-regulated in *let-7* mutants. Two conserved 6-mers complementary to let-7 mature sequence were enriched in the 3′ UTRs in the up-regulated gene set (Table S1). As expected, the nucleotides TACCTC, which are complementary to the let-7 seed sequence (nucleotides 2–7 of a mature miRNA), were enriched, consistent with the prevailing model for miRNA target recognition [42]. Also enriched was AACCTA, complementary to nucleotides 9–14 of let-7, which overlaps with the newly described “centered sites” observed for some miRNA target interactions [43]. 158 genes that were up-regulated in *let-7* mutants had at least one of these two 6-mers in their 3′ UTRs. The presence of strong seed enrichment in the up-regulated gene set led us to include an additional 8 and 5 up-regulated, predicted targets found by the seed based algorithms PicTar and TargetScan respectively, for further analysis. From the three prediction methods, there were 167 unique direct target candidates, including the known targets *lin-41*, *daf-12*, and *hbl-1*.

### Elimination of likely indirect downstream targets of *let-7* regulation

We also employed an alternative filter to select potential *let-7* targets independent of preconceptions about base pairing requirements. *let-7* is near the end of a genetic pathway controlling developmental timing in *C. elegans* [71]. Negative regulation of *lin-41* by *let-7* in late larval stages allows the transcription factor LIN-29 to accumulate and to directly control the terminal differentiation of multiple cell types [6], [7], [72], [73]. In *let-7* mutants, *lin-41* persists in late larval stages where it can continue to negatively regulate *lin-29* [6], [7]. Thus, in *let-7* mutants, larval genes turned off by *lin-29* will be up-regulated in addition to direct targets of *let-7*. In *lin-29* mutants, the same downstream larval genes should be up-regulated, yet the upstream direct targets of *let-7* should be unaffected. By analyzing gene-expression in *lin-29* versus *let-7* mutants, novel targets can be found that may not have obvious binding sites.

Three *lin-29(n333)* mutant L4 RNA samples paired with wildtype and *let-7(n2853)* samples were collected, labeled and hybridized to Affymetrix microarrays. In *lin-29(n333)*, 3030 genes were up-regulated and 1994 genes were down-regulated relative to WT samples (Table S2). Consistent with a role for *lin-29* in directing terminal differentiation and adult fates, genes up-regulated in *lin-29* mutants were enriched for GO terms for larval development (Table S2). In comparison to WT, 930 common genes were up-regulated in both *let-7(n2853)* and *lin-29(n333)* and 649 common genes were down-regulated in both. We selected the 192 genes that were up-regulated in both of the *let-7(n2853)* vs. WT and the *let-7(n2853)* vs. *lin-29(n333)* pairs, which included *lin-41*, and *daf-12*, as possible direct targets (Tables S1 and S3). Combining the candidates that emerged from the computational and mRNA expression analyses, there were 340 candidates to test for genetic interactions with *let-7*.

### Several transcription factors suppress vulval rupture in *let-7* mutants

To identify functionally important genes among the list of candidates, we used RNAi screens to find genetic interactions by suppression of *let-7* mutant phenotypes. The *let-7* mutant worms display an array of developmental timing defects at the larval to adult transition including rupturing (Rup) of the intestine and gonads through the vulva [6], [7]. The developmental defects observed in *let-7* mutants are caused by the over-expression of direct regulatory targets such as *lin-41* and *hbl-1*, and some of these defects can be suppressed by RNAi knockdown of these targets in *let-7* mutants [6], [7], [9], [10]. RNAi mediated suppression of vulval rupturing in *let-7* mutants has been used to find new genetic interactions in sets of computationally predicted targets and in genes on chromosome I [11], [38], [74]. However, many of the candidate genes from our global expression analyses have not been assayed for vulval rupture and, thus, we were able to discover novel genetic suppressors.

**Figure 2:**
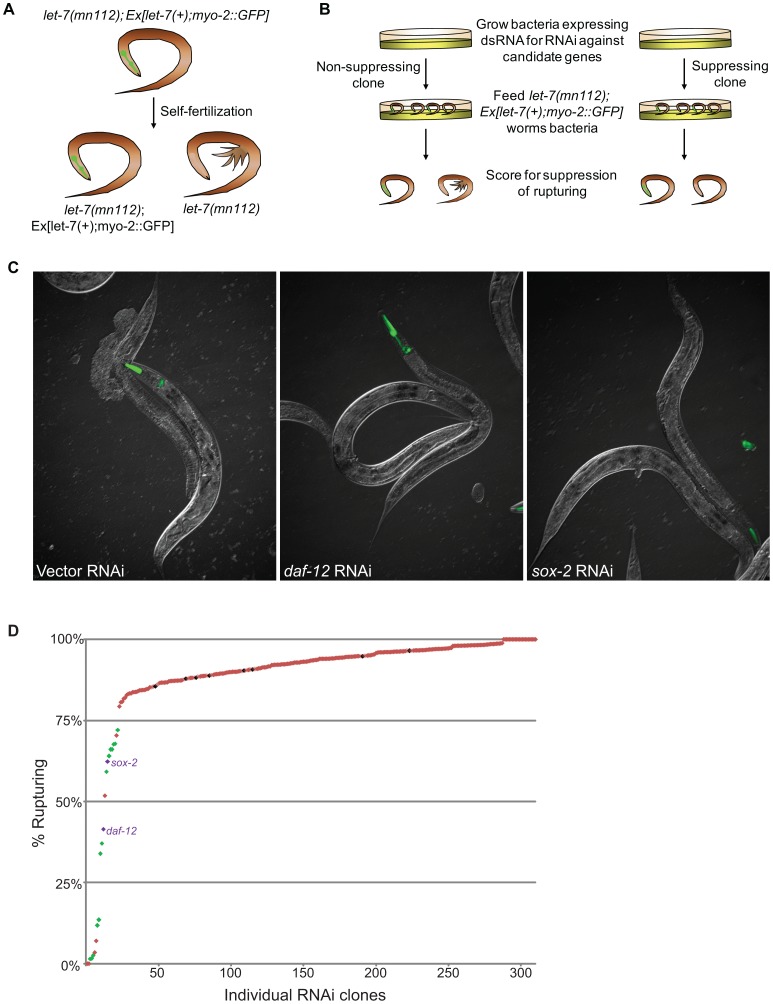
Novel suppressors of vulval rupture in *let-7* null mutants. (A) Null *let-7(mn112)* worms were maintained with an extrachromosomal rescuing transgene (let-7(+)) co-expressing a pharyngeal GFP marker (myo-2::GFP). Progeny that lack the transgene rupture from the vulva and die. (B) The *let-7(mn112); Ex[let-7(+);myo-2::GFP]* strain was grown on bacteria expressing dsRNA corresponding to candidate targets and the empty vector control. The percent rupture of non-rescued (non-GFP) animals was determined for each RNAi clone. (C) The vector control RNAi fails to suppress vulval rupturing, while knockdown of a known target (*daf-12*) or a novel candidate (*sox-2*) allows *let-7(mn112)* animals to survive to adulthood. (D) The rate of vulval rupture was plotted for each RNAi clone tested. Green points indicate clones that reduced the rupture rate to below 75% in 2/2 experiments (n>50 worms/experiment). Purple points indicate RNAi clones depicted in (C). Red points indicate clones that failed to reproducibly meet the 75% cut-off. The vector negative controls are shown in black.

Using the Ahringer feeding RNAi library [75], the Vidal feeding RNAi library [76] and a few clones we generated, 308 genes out of the 340 candidates were tested for suppression of vulval rupturing in the *let-7(mn112)* null strain. Homozygous *let-7(mn112)* mutants die at the late larval stages and must be maintained by a wild-type copy of the *let-7* gene coming from a balanced translocation or a rescuing transgene [6], [7]. To grow a population of *let-7(mn112)* mutants to be able to score suppression, we generated a transgenic strain in which the worms were maintained by the presence of an extrachromosomal array (Ex[*let-7(+); myo-2::GFP])*, which contains a *let-7* rescue fragment, allowing the mutants to survive, and the *myo-2* promoter driving expression of a GFP marker in the pharynx to indicate the presence of the array (Figure 2A). To identify new suppressors of vulval rupturing, worms were grown synchronously from the L1 stage on bacteria expressing dsRNA targeting candidate genes or empty vector, as a negative control, and populations of non-transgenic animals were scored for the rate of vulval rupturing at the late larval and young adult stages (Figure 2B–2C). Nine clones exhibited larval growth arrest and therefore could not be scored for suppression. Empty vector clones were scored eight independent times as a negative control and 86–97% of these non-rescued worms ruptured at the time of scoring. We considered clones in which less than 75% of the population exhibited rupturing as suppressors (Figure 2D), consistent with a previous screen [11]. From this, 22 suppressors were retested and 16 clones again met the suppression threshold, including known suppressors *lin-41*, *daf-12*, and *hbl-1* (Table 3) (Figure 2C–2D). Transcription factors constitute approximately half of the rupturing suppressors (7 of 16), several of which are involved in development including *fos-1*, *lin-11*, and *sox-2* [77], [78], [79], [80], [81]. Enrichment of a different set of transcription factors was also noted by the Slack lab as genetic suppressors of their computational *let-7* predictions [11].

**Table 3:** Phenotypic suppressors of let-7 mutants.

Gene	Sequence Name^1^	*let-7* fold change	*lin-29* fold change	Target prediction^2^	Phenotypes suppressed^3^	ALG-1 CLIP^4^	Gene Description
*daf-12*	F11A1.3	2.1	1.04	WPYTRGMC	R(59%)S(***)	I 3	Nuclear hormone receptor
*lin-41*	C12C8.3	2.8	1.53	W RGMC	R(98%)S(***)	C 3	Ring finger-B box-Coiled coil
*opt-2*	K04E7.2	1.15	1.18	Y	R(34%)S(***)	C	Oligopeptide transporter family member
*nhr-25*	F11C1.6	1.71	1.91	WPY	R(98%)		Nuclear hormone receptor
*hbl-1*	F13D11.2	1.23	1.07	WPYTRGMC	R(88%)	3	Zn-finger transcription factor
*adt-2*	F08C6.1	1.07	0.85		R(63%)	C 3	ADAMTS family
	T08B2.8	1.04	1.02		R(48%)		Mitochondrial ribosomal protein L23
	C26E6.6	1.13	1.09		R(36%)		Ribosomal protein L3
*lin-11*	ZC247.3	1.1	0.92		R(34%)		Homeodomain transcription factor
*sox-1*	C32E12.5	1.49	2.15	WPY R	R(66%)		HMG-box transcription factor
*sox-2*	K08A8.2	1.11	1.22	WPY M	R(38%)		HMG-box transcription factor
*fos-1*	F29G9.4	1.21	1.42	T	R(87%)		Fos bZip transcription factor family
	T25G3.3	1.15	1.11	Y	R(32%)		Ortholog of S. cerevisiae NMD3
*rha-2*	C06E1.10	1.16	1.4	Y	R(32%)		DEAH-box RNA helicase
	Y39B6A.33	1.08	1.2	Y	R(28%)		Glioma tumor suppressor candidate region gene 2
	F42A8.1	1.48	0.76		R(99%)		Unknown function
	ZK1236.1	1.13	1.08		S(*)		Elongation factor-type GTP-binding protein
*nduf-7*	W10D5.2	1.04	1.06	WP TMC	S(*)		NADH-ubiquinone oxidoreductase
*sor-1*	ZK1236.3	1.1	0.97	P	S(**)		Polycomb group-like complex member
*daf-9*	T13C5.1	1.61	1.48	PY	S(***)	3	Cytochrome P450 CYP2 subfamily
*prmt-1*	Y113G7B.17	1.19	1.29	WPY	S(**)	C 3	Protein arginine N-methyltransferase family
	T27D12.1	1.31	0.89		S(**)	C 3	Sodium/ phosphate transporter
*clec-51*	B0218.6	1.13	1.06	M	C-	Ctin fa	member

1 Sequence names from WormBase (http://www.wormbase.org).

2 W = mirWIP [41], P = PITA [40], Y = (this paper), T = TargetScan [69], R = RNA22 [39], G = MicroTarget [11], M = Miranda [36], C = PicTar [38].

3 R = Suppression of rupturing phenotype (% non-rupture), S = Suppression of the extra seam cell nuclei phenotype (significance level.

4 Locations of ALG-1 binding sites C =  coding region, I = intron, 3 = 3' UTR [66].

### *let-7*–dependent seam cell cycle exit is controlled by a diverse set of downstream genes

**Figure 3:**
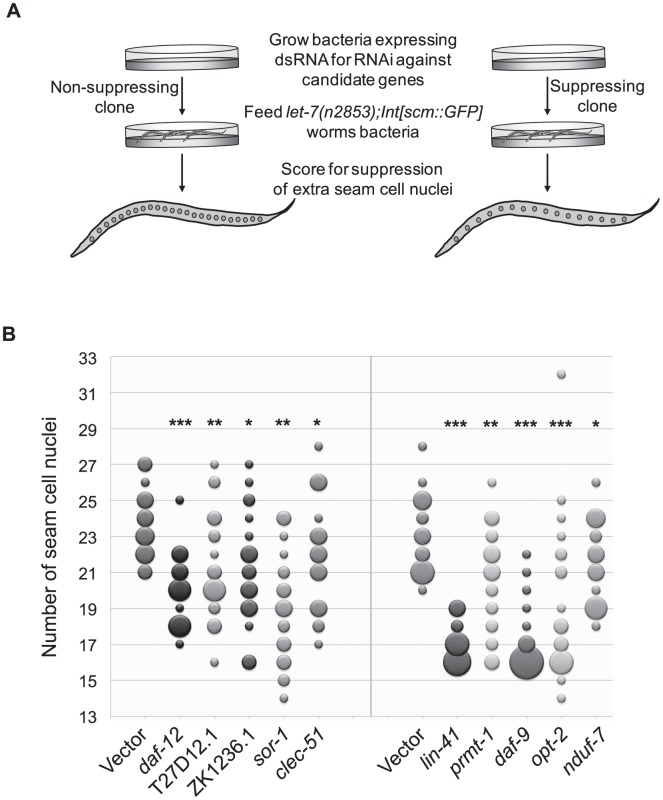
Suppression of supernumerary seam cell nuclei in *let-7* mutants. (A) While wild-type worms have 16 seam cell nuclei, *let-7(n2853)* worms have significantly more (∼23) [82]. To score for suppression of the extra seam cell phenotype, *let-7* mutants expressing nuclear GFP in seam cells (*let-7(n2853)*;*Int[scm::GFP]*) were grown at the restrictive temperature (25**°**C) on bacteria expressing dsRNA against candidate targets and the vector control. The number of seam cell nuclei was counted in a population of 20 worms evaluated against the same size population concurrently grown on the empty vector control. RNAi clones that resulted in worm populations with significantly lower seam cell numbers (p<0.05) were retested and scored using a population of at least 20 worms. (B) Suppressors of the extra seam cell nuclei phenotype in *let-7(n2853)* (p-value<0.05) are shown by bubble plot. Each bubble indicates the number of seam cell nuclei per worm for a population (n≥20) and the size of each bubble is proportional to the number of the animals in the population with a given seam cell number. * p<0.05, ** p<0.01, *** p<0.0001 in two independent trials.

To broaden the search for genes that interact with *let-7* beyond those involved in vulval rupture, we reasoned that novel targets might control other phenotypes found in *let-7* mutants. In addition to the rupturing phenotype, *let-7* mutants also have defects in the terminal differentiation of their seam cells, a specialized type of hypodermal cell [6], [7], [82]. Seam cells undergo significant changes during the larval to adult transition, including fusion of the seam cells, cessation of division, and the secretion of the adult cuticular structure known as alae [83]. Exit of the seam cells from the cell cycle and secretion of alae have been shown to be retarded in *let-7* mutants [6], [7], [82]. Interestingly, seam cell fusion was unaffected in *let-7(mn112)* null mutants, suggesting that some aspects of seam cell terminal differentiation are *let-7* independent (Figure S1). We chose to focus on the cell cycle exit defect, in which the seam cells fail to stop dividing at the larval to adult transition [82], as this would be the first screen for suppression of this phenotype and likely to uncover novel genetic interactions. Candidate RNAi clones from the rupturing suppression screen were tested for suppression of the cell cycle exit defect in *let-7(n2853)* mutants also carrying the integrated transgene *Int[scm::GFP]*, which expresses a nuclear localized GFP specifically expressed in seam cells. The number of GFP positive seam cell nuclei were counted in at least 20 young adult worms (Figure 3A). Candidates were considered suppressed if they had significantly less nuclei than empty vector grown at the same time, p<0.05 using a Mann-Whitney U test. The 23 suppressing clones yielded 10 reproducible suppressors upon retest (Figure 3B and Table 3).

Among the suppressors were *lin-41* and *daf-12*, which suppress two other *let-7* phenotypes, vulval rupture and alae formation [6], [7], [11]. Thus *lin-41* and *daf-12* RNAi are sufficient to suppress all previously described phenotypes of *let-7* mutants. Though *hbl-1* RNAi also suppresses rupturing and alae formation defects, it is not surprising that it does not suppress the extra seam cell nuclei defect because *hbl-1* loss of function mutants also have an increase in the seam cell nuclei number [10]. Of the 306 clones screened, 7 clones caused larval arrest and could not be scored. Consistent with previous work by the Gilleard lab [84] **,** *elt-1* RNAi led to the loss of most of the seam cells during larval development, rendering it inconclusive for suppression. Suppressors of the supernumerary seam cell divisions in *let-7(n2853)* represent a diverse set of gene functions and there is only modest overlap with the rupturing suppressors, suggesting that the two phenotypes are likely under separate genetic control (Table 3).

### Candidate *let-7* targets differentially affect vulva formation

**Figure 4:**
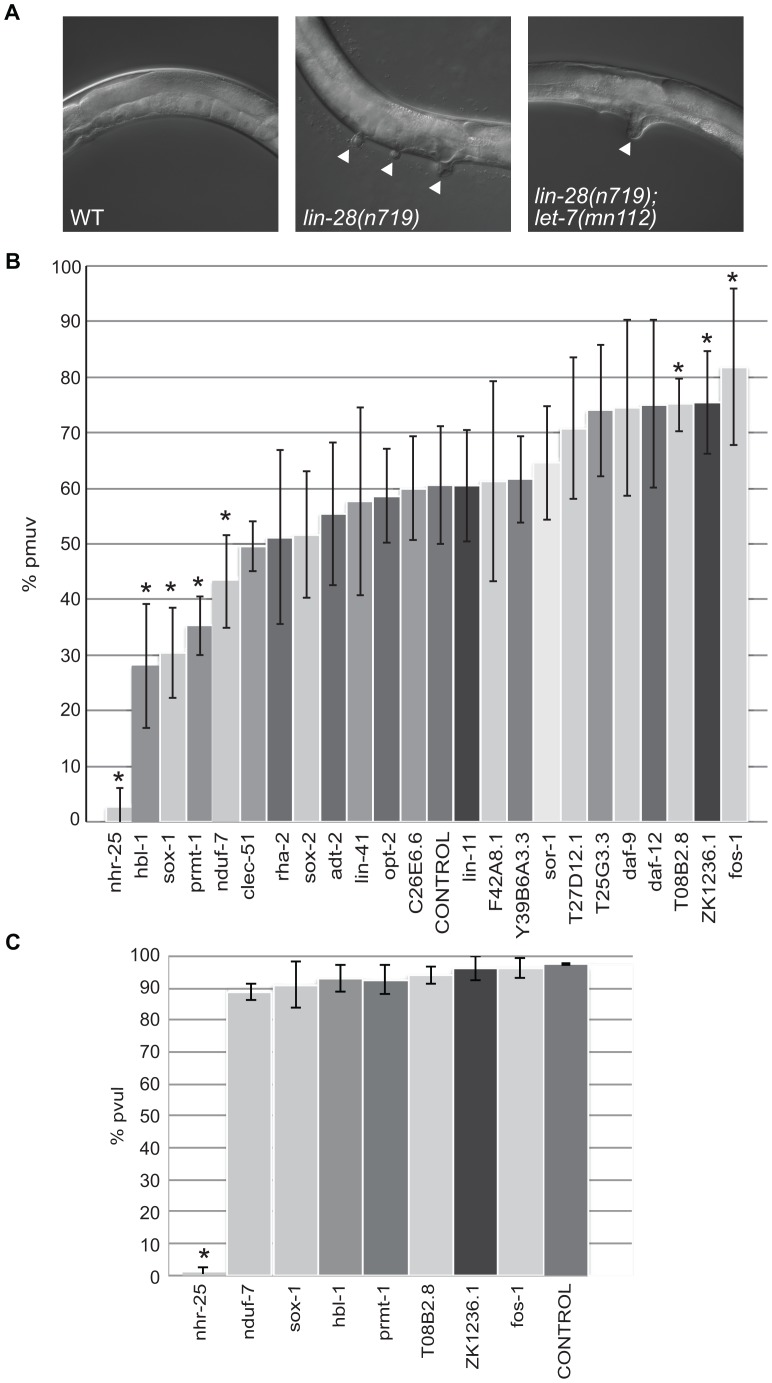
Differential effects of *let-7* target candidates on vulva formation. (A) Micrographs of the protruding multiple vulva (pmuv) phenotype in *lin-28(n719)* and the suppression to a single protruding vulva (pvul) when combined with *let-7(mn112)*. White arrowheads point to protruding vulvas in the mutants. (B) To screen for changes in the pmuv phenotype, 50–100 *lin-28(n719)* worms were grown to adulthood on vector control or gene specific RNAi plates (x-axis) and scored for percentage of pmuv (y-axis). The bar graphs represent the average percent of pmuv worms as determined from 5 independent experiments. Error bars represent the standard deviation from the mean and the * points to clones that resulted in significant enhancement or suppression in the % of pmuv worms when compared to the control (empty vector), *P<0.05. (C) To screen for changes in the pvul phenotype, 50–100 *lin-28(n719);let-7(mn112)* worms were grown to adulthood on vector control or gene specific RNAi plates (x-axis) and scored for percentage of pvul (y-axis). The bar graphs represent the average percent of pvul worms as determined from 4 independent experiments. Error bars represent the standard deviation from the mean and the * points to clones that resulted in significant suppression in the % of pvul worms when compared to the control (empty vector), *P<0.05.

The twenty-three candidate *let-7* targets were also tested for potential roles in a vulva formation abnormality due to precocious let-7 expression. The loss of function *lin-28(n719)* mutants exhibit a partially penetrant temperature-sensitive protruding multiple vulva (pmuv) phenotype that is dependent on *let-7*. At 25**°**C, this phenotype is expressed in ∼67% of the *lin-28(n719)* population with the remaining worms displaying a single protruding vulva (pvul) (Figure 4A). In the presence of the *let-7(mn112)* null allele, the pmuv phenotype is no longer observed in *lin-28(n719)* worms, and 100% of the double mutant population expresses the pvul phenotype (Figure 4A). Thus, the pmuv phenotype is dependent on *let-7*, and suggests that the precocious expression of *let-7* in the *lin-28* mutants might prematurely repress targets needed to regulate vulval cell patterning. We predicted that further suppression of such targets by RNAi would enhance the pmuv phenotype in *lin-28(n719)* worms. To identify potential targets that act in this pathway, the percent of the population exhibiting pmuv was scored for *lin-28(n719)* mutants subjected to RNAi of the 23 candidates. RNAi of three genes produced the expected enhancement of the pmuv phenotype (Figure 4B), suggesting that inappropriate down-regulation of these candidates in *lin-28* mutants contributes to mis-specification of vulval cell fates. This enhanced phenotype is dependent on *let-7* because the pmuv phenotype is almost entirely absent in *lin-28* mutant worms that also lack *let-7* activity (*lin-28(n719);let-7(mn112)*) (Figure 4C). Surprisingly, another set of genes significantly decreased the incidence of pmuv in *lin-28(n719)* (Figure 4B) and, in the case of *nhr-25*, the pvul phenotype was also suppressed in the *lin-28(n719);let-7(mn112)* double mutants (Figure 4C). These results suggest that some of the candidate genes may have a more complicated relationship with *let-7*, possibly affecting *let-7* expression or activity in tissue-specific feedback loops.

### Novel targets associated with ALG-1 in a *let-7*–dependent manner

miRNAs repress target mRNA expression through their association with Argonaute proteins allowing them to act as sequence-specific guides for the RISC complex [4], [5]. Taking advantage of the recent global map of Argonaute Like Gene 1 (ALG-1) binding sites in *C. elegans* [66], we searched for these sites in the twenty-three suppressors. Eight of the twenty-three suppressing genes had significant ALG-1 binding sites within their 3′ UTRs and coding regions. This group included the known let-7 targets, such as *daf-12* and *lin-41*, as well as *hbl-1*, which is also a target of other let-7 miRNA family members (Table 3) [7], [9], [10], [11], [85], [86], [87].

**Figure 5:**
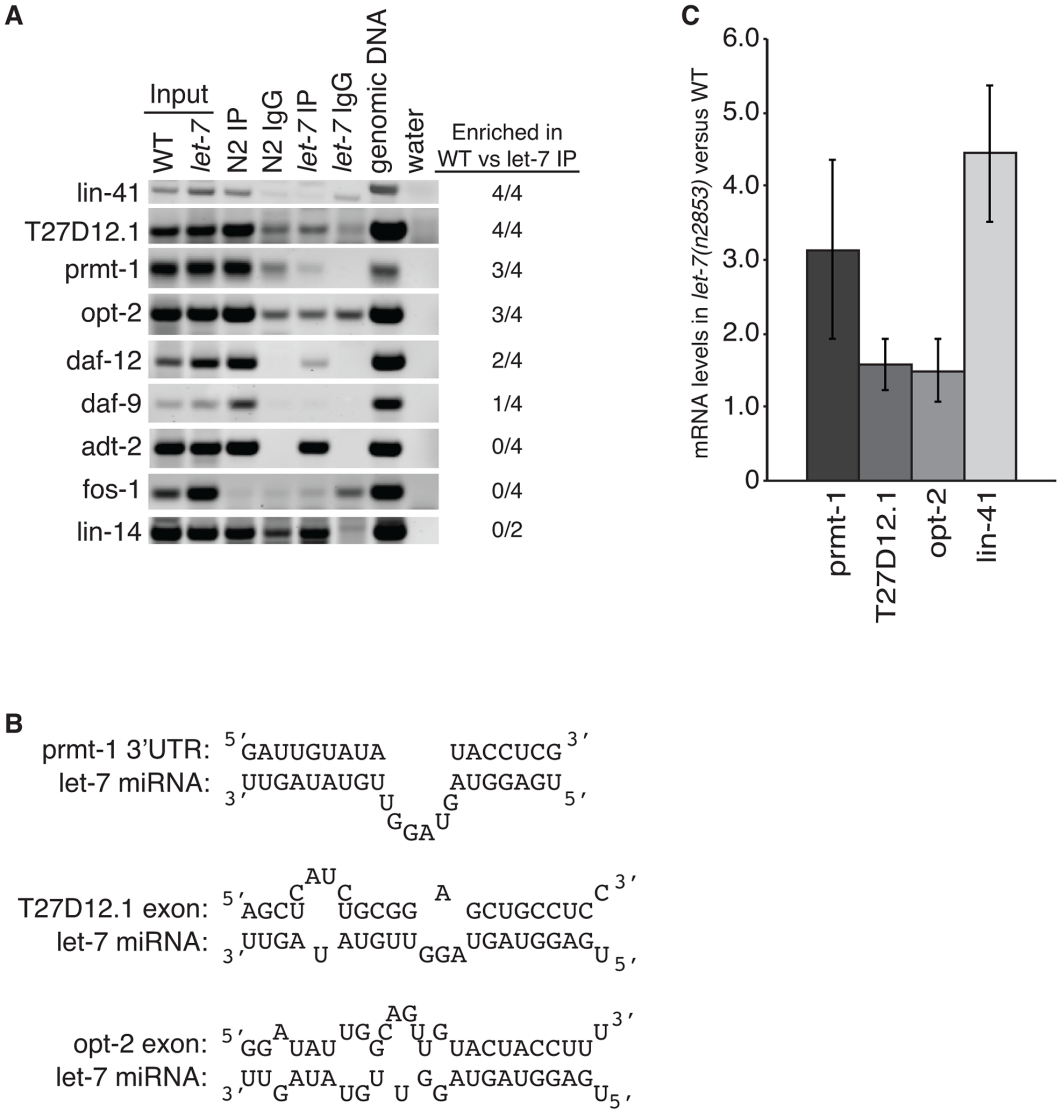
Argonaute associates with targets in a *let-7*–dependent manner. (A) Sequences in the indicated genes were detected by semi-quantitative PCR of cDNA from ALG-1 immunoprecipitation assays from L4 staged WT and *let-7(n2853)* strains. Based on enrichment in the WT compared to *let-7* RIP from 4 independent experiments, three new targets were identified, *T27D12.1*, *prmt-1*, and *opt-2*. (B) let-7 complementary sites (LCS) are present in each of the newly identified targets. Each LCS is within an ALG-1 binding site. (C) qPCR analysis of WT and *let-7(n2853)* cDNA from L4 staged worms. Targets were normalized to 18S ribosomal RNA. Shown is the average and standard deviation from 3 independent experiments.

To test if *let-7* is responsible for the interaction of ALG-1 with these genes, we analyzed their association with ALG-1 using RNA immunoprecipitation (RIP) in wild-type and *let-7(n2853)* worms (Figure 5A). Genes regulated by *let-7* are expected to be enriched in wild-type samples versus *let-7* mutant samples, while genes targeted by other miRNAs should be amplified similarly in both strains. Four independent RIPs were analyzed, and targets enriched in the wild-type for at least 2 of the 4 replicates were considered to be dependent on *let-7* for ALG-1 association. The known targets *lin-41* and *daf-12*, served as positive controls with both showing *let-7*-dependent enrichment in the ALG-1 IP. *fos-1* was used as a negative control as it did not have any significant CLIP reads nor did *fos-1* sequences amplify from the RIPs in either worm strain. *lin-14* was also used as a negative control because it is a known target of a different miRNA, lin-4, and as expected there was no significant change in ALG-1 binding in *let-7* mutants compared to WT. *daf-9* and *adt-2* had significant CLIP reads but could not be verified as targets through the RIP analysis. *adt-2* had similar levels in the WT and *let-7(n2853)* mutant strains suggesting it may be targeted by a different miRNA, which could mask any let-7 dependent RISC association.

Three novel targets were identified: *prmt-1*, *opt-2*, and T27D12.1. They were all enriched in the WT compared to the *let-7(n2853)* RIP (Figure 5A) and are, therefore, associated with ALG-1 in a let-7-dependent manner. Furthermore, we found let-7 complementary sites (LCS) within the ALG-1 binding sites of these targets (Figure 5B), supporting these genes as new direct targets of let-7. Interestingly, T27D12.1 and *opt-2*, which contain predicted target sites in coding exon sequences, showed weak mis-regulation at the mRNA level in *let-7(n2853)* versus WT worms (Figure 5C). In contrast, *prmt-1* and the positive control *lin-41*, which contain 3′UTR target sites, were up-regulated over three-fold at the mRNA level in the *let-7* mutant worms. These data are consistent with the global correlation observed between changes in mRNA levels and ALG-1 binding to 3′UTR, but not coding exon sequences [88].

## Discussion

The let-7 miRNA is exceptional in its conservation and essential role in cellular differentiation across species [13]. Loss of *let-7* activity results in lethality in worms and contributes to oncogenesis in mammalian tissues [14], [89]. Since these effects are due to mis-regulation of *let-7* targets, identification of the biologically relevant genes regulated by this miRNA has been a paramount research goal. Through a combination of genetic and molecular screens in *C. elegans*, we have uncovered twenty-three genes that are up-regulated in *let-7* mutants and contribute to the developmental abnormalities characteristic of these mutants. Three of these genes, *lin-41*, *daf-12* and *hbl-1*, are the best previously characterized *let-7* targets in *C. elegans*, validating the sensitivity of our approach [6], [7], [9], [10], [11]. Unexpectedly, a subset of the genes that suppressed *let-7* mutant phenotypes also suppressed a *lin-28* phenotype that is due to up-regulation of *let-7* expression, suggesting nonlinear pathways between these targets and *let-7* in vulval precursor cells. Three genes, *prmt-1, opt-2*, and T27D12.1, were found to associate with the miRNA complex in a *let-7* dependent manner and, thus, emerged as likely novel direct targets of *let-7*.

A large fraction of the transcriptome is mis-regulated in *let-7(n2853)* worms. Based on microarray analyses, most of these changes are less than two-fold. However, *let-7(n2853)* is a temperature sensitive loss of function strain that maintains some *let-7* activity even at the non-permissive temperatures. Accordingly, the fold change in *let-7* target mRNA expression for *lin-41*, for example, is less dramatic in *let-7(n2853)* compared to wild type at the L4 stage than it is in stages before (L2) and after (L4) *let-7* expression in wild type worms [67]. By using reproducibility in the direction of change, instead of the absolute fold difference in mRNA levels, we identified twenty new genes in the *let-7* pathway that exhibited only modest expression differences in *let-7* mutants. In fact of our list of *let-7* suppressors, only *lin-41* and *daf-12* were mis-regulated by more than two-fold by microarray analyses. The large number of down-regulated genes in *let-7(n2853)* mutants likely represents indirect targets, reflecting mis-regulation of direct targets that transcriptionally regulate some of these genes. Over one-third of the genes up and down-regulated in *let-7(n2853)* were changed in the same direction in *lin-29* mutants, indicating that failure to trigger the *lin-29-*dependent transcriptional program also accounts for many of the mis-regulated genes in *let-7* mutants.

Considering that the two well-established targets of *let-7*, *lin-41*, and *daf-12*, suppress both the rupturing vulva and extra seam cell phenotypes of *let-7* mutants, it was surprising to find almost entirely distinct sets of new genes affecting one phenotype versus the other. The *opt-2* gene was the only additional suppressor of both phenotypes, suggesting that different pathways largely control maturation of the vulva and seam cells. While it is not entirely understood why *let-7* mutants rupture through the vulva, it has been postulated that improper cell fusions during vulva formation cause weakening and destabilization of this structure. Fourteen new genes were found to suppress the bursting vulva phenotype when subjected to RNAi conditions, none of which overlapped with the previously described suppressors of this *let-7* phenotype [11], [74]. A distinction from these studies is that we screened for suppression in null *let-7(mn112)* worms as opposed to the weaker *let-7(n2853)* strain. Two of the *let-7(n2853)* suppressors identified in Grosshans et al., 2005, *lin-59* and *lss-18*, were found to be up-regulated in *let-7* mutants by our microarray analyses. However, these candidates failed to suppress the rupturing of *let-7(mn112)* worms, in agreement with the previous study [11]. Many of the genes we identified as suppressors of vulva rupturing encode transcription factors, a category also prominent on the list of potential *let-7* targets described in Grosshans et al., 2005 [11]. Genes involved in translation make up another class of *let-7(n2853)* suppressors [74]. A combined approach, incorporating *let-7* target predictions by PicTar, reporter assays and screens for suppression of rupturing in *let-7(n2853)*, resulted in twelve potential new targets [38]. Of the genes that passed the genetic test, only *fos-1* is in common with our list of bursting suppressors. Another group tested 181 genes with various criteria for being potential *let-7* targets for changes in protein levels in WT versus *let-7(n2853)* worms [54]. Of the nineteen candidates up-regulated in *let-7* mutants, nine also suppressed rupturing in *let-7(n2853)*. Three of these suppressors, *T19A6.2*, *Y47GA.10*, and *F46B6.7*, were up-regulated in our microarray data. However, they failed suppress vulva rupturing in the null *let-7(mn112)* background and, thus, did not appear on our final list of candidates. An important consideration when screening for suppression of vulva rupturing is that in some cases the effect may be indirect due to slow or halted development or the absence of vulva formation. These caveats were avoided by using the *let-7(mn112)* strain containing the extrachromosomal *let-7* rescue construct, as RNAi clones that affected development regardless of the presence of the *let-7* transgene could be flagged. Nonetheless, the observation that RNAi of many different genes results in suppression of the rupturing phenotype in *let-7* mutants points to the existence of cross-regulatory pathways that are sensitive to down-regulation of a single target.

Reiteration of seam cell nuclear divisions at the transition to adulthood is another characteristic of *let-7* mutants [11], [82]. In *C. elegans*, the lateral seam cells undergo an asymmetric division in which one daughter cell differentiates while the other repeats this pattern at each larval stage [83]. In *let-7* mutants, the seam cells inappropriately undergo the larval type division instead of differentiating to the adult fate, where the cells normally fuse and cease dividing [6]. The heterochronic gene *lin-29* is downstream of *let-7* and is a master regulator of seam cell differentiation [6], [73]. The failure of seam cells to properly differentiate in *let-7* mutants seems to be largely due to a lack of *lin-29* activity [6], [7]. How *let-7* positively regulates the expression of LIN-29 protein is presently unknown. Our screen identified eight new genes that suppress the supernumerary seam cell divisions of *let-7(n2853)* mutants. Three of these suppressors, *opt-2, prmt-1*, T27D12.1, are likely direct targets of *let-7* since their association with Argonaute is dependent on this miRNA. The group of extra seam cell suppressors includes factors with a variety of predicted functions that could potentially contribute to mis-regulation of *lin-29*.

In *C. elegans*, processing of the let-7 miRNA early in larval development is inhibited by LIN-28 protein [21], [23]. In *lin-28(n719)* mutants, let-7 miRNA is expressed precociously, resulting in premature repression of its targets. One effect of this mis-regulation is the development of protruding multiple vulvas in *lin-28* mutants grown at 25**°**C. This partially penetrant pmuv phenotype is dependent on *let-7* because *lin-28(n719);let-7(mn112)* strains only produce single protruding vulvas. Since early accumulation of let-7 miRNA is expected to cause premature down-regulation of targets, we anticipated that further silencing of potential targets by RNAi would enhance the pmuv phenotype in *lin-28(n719)* worms. Three candidates, *fos-1, ZK1236.1* and T08B2.8, emerged as enhancers, pointing to roles for these genes in vulval fate specification. Surprisingly, there were also several candidates that decreased the percentage of pmuv in *lin-28(n719)* worms including, *nhr-25*, *hbl-1*, *sox-1*, *prmt-1*, and *nduf-7*. Since this effect is also observed when *let-7* is removed from *lin-28(n719)*, these suppressors potentially feedback to regulate the expression or function of *let-7* in vulval precursor cells. Feedback loops between let-7 family members and targets, such as *daf-12* and *hbl-1*, in other tissues have been previously demonstrated [9], [10], [85], [86], [87], [90].

Multiple lines of molecular and genetic evidence support *opt-2, prmt-1* and T27D12.1 as new direct targets of *let-7* regulation. One of the targets, *opt-2*, may be a general downstream effector in the *let-7* pathway as down-regulation of *opt-2* suppresses phenotypes in the vulva and seam cells. Before this study, *opt-2* was not a predicted *let-7* target because it lacks complementarity to the 5′ end of the miRNA (seed) in its 3′UTR. However, a single ALG-1 binding site is present in the second last exon of *opt-2* and this region includes a predicted let-7 binding site. *opt-2* (also known as *pept-1*) is a member of the peptide transporter family and facilitates uptake of di- and tri-peptides in the intestine [91], [92]. Loss of *opt-2* activity slows development, alters fat accumulation and enhances stress resistance 91,93. Although *opt-2* appears to be exclusively expressed in the intestine, loss of this factor causes global changes in gene expression [94], [95]. Reporters driven by the *let-7* promoter also show intestinal expression, suggesting that let-7 miRNA is available for directly regulating *opt-2* in this tissue [96], [97], [98]. The ability of *opt-2* RNAi to suppress *let-7* phenotypes in vulval and seam cells suggests that signaling from the intestine influences development of these tissues.

Another likely direct target, T27D12.1, also seems to be regulated by let-7 through sequences in its open reading frame. This gene lacks predicted target sites for let-7 in its 3′UTR but came through our screen as a modestly up-regulated gene in *let-7(n2853)* that was capable of suppressing the extra seam cell phenotype of these mutants. T27D12.1 contains one ALG-1 binding site in its 3′UTR and one in a coding exon, but only the exonic region includes an obvious LCS, which conforms to seed-pairing with the allowance of a G-U pair. T27D12.1 is predicted to encode a sodium/phosphate transporter protein but little else is known about this factor.

The more conventional miRNA target, *prmt-1*, has an LCS within its 3′UTR and was previously predicted by the mirWIP and PITA algorithms as a let-7 target [40], [41]. While *prmt-1* has ALG-1 binding sites in its 3′UTR as well as coding exon sequences, only the 3′UTR site includes an obvious let-7 complementary site. Although there is not a canonical LCS in the 3′UTRs of mammalian homologs of *prmt-1*, there are several well conserved potential let-7 binding sites (Figure S2). *prmt-1* encodes a protein arginine methyltransferase, and it has been shown in mammalian cells to be a major contributor to methylation of histone 4 at arg-3, leading to transcriptional activation [99], [100]. Additionally, PRMT-1 has been shown to methylate arginine residues on other types of proteins in mammalian cells and *C. elegans* [93], [101]. Recently, it was discovered that PRMT-1 methylates DAF-16, a key transcription factor in the insulin pathway [101]. This modification prevents phosphorylation of DAF-16 by AKT, thus, keeping it in an active state to promote the expression of longevity-related genes. *prmt-1* has a broad expression pattern that is largely overlapping with *let-7* transcriptional reporters [96], [97], [98], [101]. Down-regulation of *prmt-1* by *let-7* in late larval stages could influence the lifespan of worms by causing reduced methylation and, hence, activity of DAF-16.

Our combination of molecular and genetic screens revealed a complex network of genes that interact with *let-7* in *C. elegans*. This approach was sensitive enough to detect the established *let-7* targets, *lin-41*, *daf-12* and *hbl-1*. While these genes are regulated at the mRNA level, other targets that are only subject to translational repression would be missed by focusing on transcripts up-regulated in *let-7* mutants. However, the microarray data revealed that thousands of genes are mis-regulated when there is insufficient *let-7* activity, supporting a widespread role for this miRNA in regulating, directly and indirectly, gene expression. A set of the up-regulated genes proved to be biologically relevant for the developmental abnormalities that arise in the absence of *let-7* activity. At least three of these genes, which encode transport proteins and a modifying enzyme, appear to be new direct targets of *let-7*. In conclusion, *let-7* appears to regulate a variety of direct targets, which in turn influences the expression of hundreds of other genes. Loss of this miRNA alone results in extensive changes in gene expression and abnormal development in multiple tissues, supporting the role of *let-7* as a master gene regulator.

## Materials and Methods

### Nematode strains

The *C. elegans* strains were cultured at 15°C or 25°C under standard conditions [102]. Worms were synchronized by hypochlorite treatment and development was initiated by plating arrested L1 hatchlings on NGM plates seeded with OP50 bacteria or RNAi bacteria on RNAi plates. Strains used in this study include the following: wild type (WT) Bristol N2, MT7626 *let-7(n2853)*, MT333 *lin-29(n333)*, MT1524 *lin-28(n719)*, PQ79 mnDp1(X/V)/+; *unc-3(ed151) let-7(mn112); Ex[let-7(+); myo-2::GFP]*, PQ270 mnDp1(X/V)/+; *unc-3(ed151) let-7(mn112)*; *lin-28(n719)*, PQ293 *let-7(n2853); Int[scm::GFP]*.

### RNAi experiments

Seam cell nuclei were counted at 40 hr (25°C) in 20 adult PQ293 *let-7(n2853); Int[scm::GFP]* worms grown on vector control or gene specific RNAi plates for one generation. Suppression was determined by a Mann-Whitney U test comparing worms on each RNAi vector to those on the empty L4440 control vector grown on the same day. Bursting suppression was scored as more than 25% non-bursting, non-green (non-rescued) 40 hr adult PQ79 mnDp1(X/V)/+; *unc-3(ed151) let-7(mn112); Ex[let-7(+); myo-2::GFP]* worms grown at 25°C. All suppressing clones were retested using the same criteria for reproducibility. All clones suppressing at least one phenotype were verified by sequencing. Fifty to one hundred *lin-28(n719)* or *lin-28(n719);let-7(mn112)* worms were grown on RNAi until 48 hr (25°C) adults and then scored for the protruding multivulva (Pmuv) or protruding single vulva (Pvul) phenotypes. Suppression/enhancement was determined by a Ttest comparing worms on each RNAi clone to those on the empty L4440 control vector grown at the same time for 4 or 5 independent RNAi experiments.

### Microarray analysis

Six paired replicates of L4 RNA from WT or *let-7(n2853)* worms were prepared and labeled as per manufacturer's instructions (Affymetrix, Santa Clara) and hybridized to Affymetrix *C. elegans* Gene microarrays. Three of the paired replicates of WT and *let-7(n2853)* were also paired with *lin-29(n333)* replicates for array analysis. To assess the significance of differential gene expression between the two groups, a paired t-statistic was computed. CEL files obtained after scanning were analyzed by using Affymetrix APT tools and Robust Multi-array Average (RMA)-sketch normalized [103]. Annotation files for the probe sets were obtained from Affymetrix. The paired t-test statistic was utilized to compute differences between groups for each probe set. Probe sets were mapped to custom gene structures generated from Refseq annotations obtained from ce2 at the UCSC genome browser. Gene ontology analyses were performed using the database for annotation, visualization and integrated discovery (DAVID) and the Functional Annotation Clustering Tool [104], [105]. Classifications were set to the highest stringency and the recommended enrichment score of ≥1.3 was applied. To search for enriched motifs in the gene lists, pair-wise alignments between *C. briggsae* (cb1) and *C. elegans* (ce2) were obtained from the UCSC genome browser. 3′UTR exons were spliced together to generate the sequence if necessary, and then extended to 2000 bases from the stop codon. 6-mer enrichment in genes up-regulated in *let-7(n2853)* versus non-regulated genes was computed using methods described in [106].

### RNA immunoprecipitation (RIP)

RIP assays were preformed as previously described [23], [88]. Synchronized WT and *let-7(n2853)* worms were grown at 25°C for 29 hours before being cross-linked by UV treatment. Equal amounts of lysates were pre-cleared before immunoprecipitation with the anti-ALG-1 antibody (Thermo Fisher Scientific) or control IgG (Caltag Laboratories) and protein G Dynabeads (Invitrogen). Immunoprecipitated material was subjected to Proteinase K treatment and RNA extraction before reverse transcription using random oligo priming. The resulting cDNA was used in PCR with the primers listed in Table S4.

### qPCR

RNA was isolated from WT and *let-7(n2853)* worms grown at 25°C for 28 hours. qPCR was performed on cDNA with SYBR green (Applied Biosystems) and 10 uM of each forward and reverse primer on an ABI Prism 7000 real time PCR machine. Primers are listed in Table S4.

## Supporting Information

Figure S1 Seam cell fusion proceeds normally in *let-7* mutants. Indirect immunofluorescence with the MH27 monoclonal α-AJM-1 antibody labels apical junctions in hypodermal cells, allowing visualization of seam cell fusion. Fused seam cells are seen in WT (A) and *let-7(mn112)* (B) at the young adult stage by the lack of junctions between cells (white arrowheads), which are apparent in *lin-29(n333)* worms where seam cell fusion fails (C). No dramatic decrease in fusion was seen in either *let-7(mn112)* early/mid L4 worms with (*let-7(mn112); Ex[let-7(+);myo-2::GFP]*) or without (*let-7(mn112)*) the rescue fragment (D) or in *let-7(n2853)* compared to WT worms at the adult stage (E).
(DOCX)

Figure S2 Conservation of potential let-7 complementary sites (LCSs) in mammalian prmt-1. (A) Genome browser track showing the last exon of PRMT1. Base-wise conservation from PhastCons is shown in green for all species with available alignments on the UCSC Genome Browser. 3′UTR locations complementary to let-7 are drawn as black rectangles. (B) PRMT1::let-7 duplexes predicted by RNAhybrid. Capital letters denote paired bases, lower-case letters indicate unpaired bases, dashes indicate gaps. Minimum free energy of binding is listed on the right side of each duplex. (C) All five LCS positions and their conservation across the available genome alignments is shown. Dots indicate an exact match to the human reference. Vertical orange lines indicate insertions or deletions with the size of gaps listed along the bottom of each alignment.
(DOCX)

Table S1 Differential gene expression in *let-7(n2853)* versus N2 wildtype worms. Sheet 1 shows the microarray results of mRNA expression in *let-7(n2853)* versus N2 wildtype worms at the L4 stage. Sheets 2 and 3 show the results of DAVID analysis for genes up- or down-regulated in *let-7(n2853)*, respectively. Sheet 4 indicates ALG-1 binding sites in candidates selected for phenotypic assays. Sheet 5 lists the enriched motifs found in the 3′UTRs of genes up-regulated in *let-7(n2853)*.
(XLSX)

Table S2 Differential gene expression in *lin-29(n333)* versus N2 wildtype worms. Sheet 1 shows the microarray results of mRNA expression in *lin-29(n333)* versus N2 wildtype worms at the L4 stage. Sheets 2 and 3 show the results of DAVID analysis for genes up- or down-regulated in *lin-29(n333)*, respectively.
(XLS)

Table S3 Differential gene expression in *let-7(n2853)* versus *lin-29(n333)* worms. Sheet 1 shows the microarray results of mRNA expression in *let-7(n2853)* versus *lin-29(n333)* worms at the L4 stage. Sheets 2 and 3 show the results of DAVID analysis for genes up- or down-regulated in *let-7(n2853)* versus *lin-29(n333)*, respectively.
(XLS)

Table S4 List of primer sequences used in this study.
(XLSX)

## References

[1] Aalto AP, Pasquinelli AE (2012) Small non-coding RNAs mount a silent revolution in gene expression. Curr Opin in Cell Biol 24 (3) 333–40 doi:10.1016/j.ceb.2012.03.006. 22464106 10.1016/j.ceb.2012.03.006 PMC3372702

[2] Kim VN, Han J, Siomi MC (2009) Biogenesis of small RNAs in animals. Nature reviews Molecular cell biology 10: 126–139. 19165215 10.1038/nrm2632

[3] Winter J, Jung S, Keller S, Gregory RI, Diederichs S (2009) Many roads to maturity: microRNA biogenesis pathways and their regulation. Nature cell biology 11: 228–234. 19255566 10.1038/ncb0309-228

[4] Huntzinger E, Izaurralde E (2011) Gene silencing by microRNAs: contributions of translational repression and mRNA decay. Nature reviews Genetics 12: 99–110. 10.1038/nrg2936 21245828

[5] Pasquinelli AE (2012) MicroRNAs and their targets: recognition, regulation and an emerging reciprocal relationship. Nature reviews Genetics 13: 271–282. 10.1038/nrg3162 22411466

[6] Reinhart BJ, Slack FJ, Basson M, Pasquinelli AE, Bettinger JC, et al (2000) The 21-nucleotide let-7 RNA regulates developmental timing in Caenorhabditis elegans. Nature 403: 901–906. 10706289 10.1038/35002607

[7] Slack FJ, Basson M, Liu Z, Ambros V, Horvitz HR, et al (2000) The lin-41 RBCC gene acts in the C. elegans heterochronic pathway between the let-7 regulatory RNA and the LIN-29 transcription factor. Molecular cell 5: 659–669. 10882102 10.1016/s1097-2765(00)80245-2

[8] Ambros V, Horvitz HR (1984) Heterochronic mutants of the nematode Caenorhabditis elegans. Science 226: 409–416. 6494891 10.1126/science.6494891

[9] Abrahante JE, Daul AL, Li M, Volk ML, Tennessen JM, et al (2003) The Caenorhabditis elegans hunchback-like gene lin-57/hbl-1 controls developmental time and is regulated by microRNAs. Developmental cell 4: 625–637. 12737799 10.1016/s1534-5807(03)00127-8

[10] Lin SY, Johnson SM, Abraham M, Vella MC, Pasquinelli A, et al (2003) The C elegans hunchback homolog, hbl-1, controls temporal patterning and is a probable microRNA target. Developmental cell 4: 639–650. 12737800 10.1016/s1534-5807(03)00124-2

[11] Grosshans H, Johnson T, Reinert KL, Gerstein M, Slack FJ (2005) The temporal patterning microRNA let-7 regulates several transcription factors at the larval to adult transition in C. elegans. Developmental cell 8: 321–330. 15737928 10.1016/j.devcel.2004.12.019

[12] Pasquinelli AE, Reinhart BJ, Slack F, Martindale MQ, Kuroda MI, et al (2000) Conservation of the sequence and temporal expression of let-7 heterochronic regulatory RNA. Nature 408: 86–89. 11081512 10.1038/35040556

[13] Mondol V, Pasquinelli AE (2012) Let's make it happen: the role of let-7 microRNA in development. Current topics in developmental biology 99: 1–30. 22365733 10.1016/B978-0-12-387038-4.00001-X

[14] Boyerinas B, Park SM, Hau A, Murmann AE, Peter ME (2010) The role of let-7 in cell differentiation and cancer. Endocrine-related cancer 17: F19–36. 19779035 10.1677/ERC-09-0184

[15] Thornton JE, Gregory RI (2012) How does Lin28 let-7 control development and disease? Trends Cell Biol 474–82 doi:10.1016/j.tcb.2012.06.001. 22784697 10.1016/j.tcb.2012.06.001 PMC3432650

[16] Heo I, Joo C, Cho J, Ha M, Han J, et al (2008) Lin28 mediates the terminal uridylation of let-7 precursor MicroRNA. Molecular cell 32: 276–284. 18951094 10.1016/j.molcel.2008.09.014

[17] Newman MA, Thomson JM, Hammond SM (2008) Lin-28 interaction with the Let-7 precursor loop mediates regulated microRNA processing. RNA 14: 1539–1549. 18566191 10.1261/rna.1155108 PMC2491462

[18] Rybak A, Fuchs H, Smirnova L, Brandt C, Pohl EE, et al (2008) A feedback loop comprising lin-28 and let-7 controls pre-let-7 maturation during neural stem-cell commitment. Nature cell biology 10: 987–993. 18604195 10.1038/ncb1759

[19] Viswanathan SR, Daley GQ, Gregory RI (2008) Selective blockade of microRNA processing by Lin28. Science 320: 97–100. 18292307 10.1126/science.1154040 PMC3368499

[20] Heo I, Joo C, Kim YK, Ha M, Yoon MJ, et al (2009) TUT4 in concert with Lin28 suppresses microRNA biogenesis through pre-microRNA uridylation. Cell 138: 696–708. 19703396 10.1016/j.cell.2009.08.002

[21] Lehrbach NJ, Armisen J, Lightfoot HL, Murfitt KJ, Bugaut A, et al (2009) LIN-28 and the poly(U) polymerase PUP-2 regulate let-7 microRNA processing in Caenorhabditis elegans. Nature structural & molecular biology 16: 1016–1020. 10.1038/nsmb.1675 PMC2988485 19713957

[22] Piskounova E, Polytarchou C, Thornton JE, LaPierre RJ, Pothoulakis C, et al (2011) Lin28A and Lin28B inhibit let-7 microRNA biogenesis by distinct mechanisms. Cell 147: 1066–1079. 22118463 10.1016/j.cell.2011.10.039 PMC3227872

[23] Van Wynsberghe PM, Kai ZS, Massirer KB, Burton VH, Yeo GW, et al (2011) LIN-28 co-transcriptionally binds primary let-7 to regulate miRNA maturation in Caenorhabditis elegans. Nature structural & molecular biology 18: 302–308. 10.1038/nsmb.1986 PMC3077891 21297634

[24] Viswanathan SR, Powers JT, Einhorn W, Hoshida Y, Ng TL, et al (2009) Lin28 promotes transformation and is associated with advanced human malignancies. Nature genetics 41: 843–848. 19483683 10.1038/ng.392 PMC2757943

[25] Zhu H, Shyh-Chang N, Segre AV, Shinoda G, Shah SP, et al (2011) The Lin28/let-7 axis regulates glucose metabolism. Cell 147: 81–94. 21962509 10.1016/j.cell.2011.08.033 PMC3353524

[26] Frost RJ, Olson EN (2011) Control of glucose homeostasis and insulin sensitivity by the Let-7 family of microRNAs. Proceedings of the National Academy of Sciences of the United States of America 108: 21075–21080. 22160727 10.1073/pnas.1118922109 PMC3248488

[27] Johnson SM, Grosshans H, Shingara J, Byrom M, Jarvis R, et al (2005) RAS is regulated by the let-7 microRNA family. Cell 120: 635–647. 15766527 10.1016/j.cell.2005.01.014

[28] Johnson CD, Esquela-Kerscher A, Stefani G, Byrom M, Kelnar K, et al (2007) The let-7 microRNA represses cell proliferation pathways in human cells. Cancer research 67: 7713–7722. 17699775 10.1158/0008-5472.CAN-07-1083

[29] Boyerinas B, Park SM, Shomron N, Hedegaard MM, Vinther J, et al (2008) Identification of let-7-regulated oncofetal genes. Cancer research 68: 2587–2591. 18413726 10.1158/0008-5472.CAN-08-0264

[30] Shell S, Park SM, Radjabi AR, Schickel R, Kistner EO, et al (2007) Let-7 expression defines two differentiation stages of cancer. Proceedings of the National Academy of Sciences of the United States of America 104: 11400–11405. 17600087 10.1073/pnas.0704372104 PMC2040910

[31] Yu F, Yao H, Zhu P, Zhang X, Pan Q, et al (2007) let-7 regulates self renewal and tumorigenicity of breast cancer cells. Cell 131: 1109–1123. 18083101 10.1016/j.cell.2007.10.054

[32] Lee YS, Dutta A (2007) The tumor suppressor microRNA let-7 represses the HMGA2 oncogene. Genes & development 21: 1025–1030. 17437991 10.1101/gad.1540407 PMC1855228

[33] Mayr C, Hemann MT, Bartel DP (2007) Disrupting the pairing between let-7 and Hmga2 enhances oncogenic transformation. Science 315: 1576–1579. 17322030 10.1126/science.1137999 PMC2556962

[34] Esquela-Kerscher A, Trang P, Wiggins JF, Patrawala L, Cheng A, et al (2008) The let-7 microRNA reduces tumor growth in mouse models of lung cancer. Cell cycle 7: 759–764. 18344688 10.4161/cc.7.6.5834

[35] Kumar MS, Erkeland SJ, Pester RE, Chen CY, Ebert MS, et al (2008) Suppression of non-small cell lung tumor development by the let-7 microRNA family. Proceedings of the National Academy of Sciences of the United States of America 105: 3903–3908. 18308936 10.1073/pnas.0712321105 PMC2268826

[36] Enright AJ, John B, Gaul U, Tuschl T, Sander C, et al (2003) MicroRNA targets in Drosophila. Genome biology 5: R1. 14709173 10.1186/gb-2003-5-1-r1 PMC395733

[37] Lewis BP, Shih IH, Jones-Rhoades MW, Bartel DP, Burge CB (2003) Prediction of mammalian microRNA targets. Cell 115: 787–798. 14697198 10.1016/s0092-8674(03)01018-3

[38] Lall S, Grun D, Krek A, Chen K, Wang YL, et al (2006) A genome-wide map of conserved microRNA targets in C. elegans. Current biology : CB 16: 460–471. 16458514 10.1016/j.cub.2006.01.050

[39] Miranda KC, Huynh T, Tay Y, Ang YS, Tam WL, et al (2006) A pattern-based method for the identification of MicroRNA binding sites and their corresponding heteroduplexes. Cell 126: 1203–1217. 16990141 10.1016/j.cell.2006.07.031

[40] Kertesz M, Iovino N, Unnerstall U, Gaul U, Segal E (2007) The role of site accessibility in microRNA target recognition. Nature genetics 39: 1278–1284. 17893677 10.1038/ng2135

[41] Hammell M, Long D, Zhang L, Lee A, Carmack CS, et al (2008) mirWIP: microRNA target prediction based on microRNA-containing ribonucleoprotein-enriched transcripts. Nature methods 5: 813–819. 19160516 10.1038/nmeth.1247 PMC3092588

[42] Bartel DP (2009) MicroRNAs: target recognition and regulatory functions. Cell 136: 215–233. 19167326 10.1016/j.cell.2009.01.002 PMC3794896

[43] Shin C, Nam JW, Farh KK, Chiang HR, Shkumatava A, et al (2010) Expanding the microRNA targeting code: functional sites with centered pairing. Molecular cell 38: 789–802. 20620952 10.1016/j.molcel.2010.06.005 PMC2942757

[44] Rigoutsos I (2009) New tricks for animal microRNAS: targeting of amino acid coding regions at conserved and nonconserved sites. Cancer research 69: 3245–3248. 19351814 10.1158/0008-5472.CAN-09-0352

[45] Wightman B, Ha I, Ruvkun G (1993) Posttranscriptional regulation of the heterochronic gene lin-14 by lin-4 mediates temporal pattern formation in C. elegans. Cell 75: 855–862. 8252622 10.1016/0092-8674(93)90530-4

[46] Lee RC, Feinbaum RL, Ambros V (1993) The C. elegans heterochronic gene lin-4 encodes small RNAs with antisense complementarity to lin-14. Cell 75: 843–854. 8252621 10.1016/0092-8674(93)90529-y

[47] Thomson DW, Bracken CP, Goodall GJ (2011) Experimental strategies for microRNA target identification. Nucleic acids research 39: 6845–6853. 21652644 10.1093/nar/gkr330 PMC3167600

[48] Huang JC, Babak T, Corson TW, Chua G, Khan S, et al (2007) Using expression profiling data to identify human microRNA targets. Nature methods 4: 1045–1049. 18026111 10.1038/nmeth1130

[49] Lim LP, Lau NC, Garrett-Engele P, Grimson A, Schelter JM, et al (2005) Microarray analysis shows that some microRNAs downregulate large numbers of target mRNAs. Nature 433: 769–773. 15685193 10.1038/nature03315

[50] Schmitter D, Filkowski J, Sewer A, Pillai RS, Oakeley EJ, et al (2006) Effects of Dicer and Argonaute down-regulation on mRNA levels in human HEK293 cells. Nucleic acids research 34: 4801–4815. 16971455 10.1093/nar/gkl646 PMC1635286

[51] Sood P, Krek A, Zavolan M, Macino G, Rajewsky N (2006) Cell-type-specific signatures of microRNAs on target mRNA expression. Proceedings of the National Academy of Sciences of the United States of America 103: 2746–2751. 16477010 10.1073/pnas.0511045103 PMC1413820

[52] Baek D, Villen J, Shin C, Camargo FD, Gygi SP, et al (2008) The impact of microRNAs on protein output. Nature 455: 64–71. 18668037 10.1038/nature07242 PMC2745094

[53] Selbach M, Schwanhausser B, Thierfelder N, Fang Z, Khanin R, et al (2008) Widespread changes in protein synthesis induced by microRNAs. Nature 455: 58–63. 18668040 10.1038/nature07228

[54] Jovanovic M, Reiter L, Picotti P, Lange V, Bogan E, et al (2010) A quantitative targeted proteomics approach to validate predicted microRNA targets in C. elegans. Nature methods 7: 837–842. 20835247 10.1038/nmeth.1504 PMC3444237

[55] Guo H, Ingolia NT, Weissman JS, Bartel DP (2010) Mammalian microRNAs predominantly act to decrease target mRNA levels. Nature 466: 835–840. 20703300 10.1038/nature09267 PMC2990499

[56] Stadler M, Artiles K, Pak J, Fire A (2012) Contributions of mRNA abundance, ribosome loading, and post- or peri-translational effects to temporal repression of C. elegans heterochronic miRNA targets. Genome Res 22 (12) 2418–26 doi:10.1101/gr.136515.111. 22855835 10.1101/gr.136515.111 PMC3514671

[57] Beitzinger M, Peters L, Zhu JY, Kremmer E, Meister G (2007) Identification of human microRNA targets from isolated argonaute protein complexes. RNA biology 4: 76–84. 17637574 10.4161/rna.4.2.4640

[58] Easow G, Teleman AA, Cohen SM (2007) Isolation of microRNA targets by miRNP immunopurification. RNA 13: 1198–1204. 17592038 10.1261/rna.563707 PMC1924889

[59] Hendrickson DG, Hogan DJ, Herschlag D, Ferrell JE, Brown PO (2008) Systematic identification of mRNAs recruited to argonaute 2 by specific microRNAs and corresponding changes in transcript abundance. PLoS ONE 3: e2126 doi:10.1371/journal.pone.0002126. 18461144 10.1371/journal.pone.0002126 PMC2330160

[60] Karginov FV, Conaco C, Xuan Z, Schmidt BH, Parker JS, et al (2007) A biochemical approach to identifying microRNA targets. Proceedings of the National Academy of Sciences of the United States of America 104: 19291–19296. 18042700 10.1073/pnas.0709971104 PMC2148283

[61] Landthaler M, Gaidatzis D, Rothballer A, Chen PY, Soll SJ, et al (2008) Molecular characterization of human Argonaute-containing ribonucleoprotein complexes and their bound target mRNAs. RNA 14: 2580–2596. 18978028 10.1261/rna.1351608 PMC2590962

[62] Zhang L, Ding L, Cheung TH, Dong MQ, Chen J, et al (2007) Systematic identification of C. elegans miRISC proteins, miRNAs, and mRNA targets by their interactions with GW182 proteins AIN-1 and AIN-2. Molecular cell 28: 598–613. 18042455 10.1016/j.molcel.2007.09.014 PMC2186060

[63] Chi SW, Zang JB, Mele A, Darnell RB (2009) Argonaute HITS-CLIP decodes microRNA-mRNA interaction maps. Nature 460: 479–486. 19536157 10.1038/nature08170 PMC2733940

[64] Hafner M, Landthaler M, Burger L, Khorshid M, Hausser J, et al (2010) Transcriptome-wide identification of RNA-binding protein and microRNA target sites by PAR-CLIP. Cell 141: 129–141. 20371350 10.1016/j.cell.2010.03.009 PMC2861495

[65] Leung AK, Young AG, Bhutkar A, Zheng GX, Bosson AD, et al (2011) Genome-wide identification of Ago2 binding sites from mouse embryonic stem cells with and without mature microRNAs. Nature structural & molecular biology 18: 237–244. 10.1038/nsmb.1991 PMC3078052 21258322

[66] Zisoulis DG, Lovci MT, Wilbert ML, Hutt KR, Liang TY, et al (2010) Comprehensive discovery of endogenous Argonaute binding sites in Caenorhabditis elegans. Nature structural & molecular biology 17: 173–179. 10.1038/nsmb.1745 PMC2834287 20062054

[67] Bagga S, Bracht J, Hunter S, Massirer K, Holtz J, et al (2005) Regulation by let-7 and lin-4 miRNAs results in target mRNA degradation. Cell 122: 553–563. 16122423 10.1016/j.cell.2005.07.031

[68] Ding XC, Grosshans H (2009) Repression of C. elegans microRNA targets at the initiation level of translation requires GW182 proteins. The EMBO journal 28: 213–222. 19131968 10.1038/emboj.2008.275 PMC2637332

[69] Lewis BP, Burge CB, Bartel DP (2005) Conserved seed pairing, often flanked by adenosines, indicates that thousands of human genes are microRNA targets. Cell 120: 15–20. 15652477 10.1016/j.cell.2004.12.035

[70] Vella MC, Choi EY, Lin SY, Reinert K, Slack FJ (2004) The C. elegans microRNA let-7 binds to imperfect let-7 complementary sites from the lin-41 3′UTR. Genes & development 18: 132–137. 14729570 10.1101/gad.1165404 PMC324419

[71] Nimmo RA, Slack FJ (2009) An elegant miRror: microRNAs in stem cells, developmental timing and cancer. Chromosoma 118: 405–418. 19340450 10.1007/s00412-009-0210-z PMC4322900

[72] Bettinger JC, Lee K, Rougvie AE (1996) Stage-specific accumulation of the terminal differentiation factor LIN-29 during Caenorhabditis elegans development. Development 122: 2517–2527. 8756296 10.1242/dev.122.8.2517

[73] Rougvie AE, Ambros V (1995) The heterochronic gene lin-29 encodes a zinc finger protein that controls a terminal differentiation event in Caenorhabditis elegans. Development 121: 2491–2500. 7671813 10.1242/dev.121.8.2491

[74] Ding XC, Slack FJ, Grosshans H (2008) The let-7 microRNA interfaces extensively with the translation machinery to regulate cell differentiation. Cell cycle 7: 3083–3090. 18818519 10.4161/cc.7.19.6778 PMC2887667

[75] Kamath RS, Fraser AG, Dong Y, Poulin G, Durbin R, et al (2003) Systematic functional analysis of the Caenorhabditis elegans genome using RNAi. Nature 421: 231–237. 12529635 10.1038/nature01278

[76] Rual JF, Ceron J, Koreth J, Hao T, Nicot AS, et al (2004) Toward improving Caenorhabditis elegans phenome mapping with an ORFeome-based RNAi library. Genome research 14: 2162–2168. 15489339 10.1101/gr.2505604 PMC528933

[77] Sternberg PW (2005) Vulval development. WormBook : the online review of C elegans biology 1–28. 18050418 10.1895/wormbook.1.6.1 PMC4781130

[78] Sherwood DR, Butler JA, Kramer JM, Sternberg PW (2005) FOS-1 promotes basement-membrane removal during anchor-cell invasion in C. elegans. Cell 121: 951–962. 15960981 10.1016/j.cell.2005.03.031

[79] Mohamadnejad M, Swenson ES (2008) Induced pluripotent cells mimicking human embryonic stem cells. Archives of Iranian medicine 11: 125–128. 18154436

[80] Yu J, Vodyanik MA, Smuga-Otto K, Antosiewicz-Bourget J, Frane JL, et al (2007) Induced pluripotent stem cell lines derived from human somatic cells. Science 318: 1917–1920. 18029452 10.1126/science.1151526

[81] Tay Y, Zhang J, Thomson AM, Lim B, Rigoutsos I (2008) MicroRNAs to Nanog, Oct4 and Sox2 coding regions modulate embryonic stem cell differentiation. Nature 455: 1124–1128. 18806776 10.1038/nature07299

[82] Hayes GD, Frand AR, Ruvkun G (2006) The mir-84 and let-7 paralogous microRNA genes of Caenorhabditis elegans direct the cessation of molting via the conserved nuclear hormone receptors NHR-23 and NHR-25. Development 133: 4631–4641. 17065234 10.1242/dev.02655

[83] Sulston JE, Horvitz HR (1977) Post-embryonic cell lineages of the nematode, Caenorhabditis elegans. Developmental biology 56: 110–156. 838129 10.1016/0012-1606(77)90158-0

[84] Smith JA, McGarr P, Gilleard JS (2005) The Caenorhabditis elegans GATA factor elt-1 is essential for differentiation and maintenance of hypodermal seam cells and for normal locomotion. Journal of cell science 118: 5709–5719. 16303852 10.1242/jcs.02678

[85] Abbott AL, Alvarez-Saavedra E, Miska EA, Lau NC, Bartel DP, et al (2005) The let-7 MicroRNA family members mir-48, mir-84, and mir-241 function together to regulate developmental timing in Caenorhabditis elegans. Developmental cell 9: 403–414. 16139228 10.1016/j.devcel.2005.07.009 PMC3969732

[86] Bethke A, Fielenbach N, Wang Z, Mangelsdorf DJ, Antebi A (2009) Nuclear hormone receptor regulation of microRNAs controls developmental progression. Science 324: 95–98. 19342589 10.1126/science.1164899 PMC2757405

[87] Hammell CM, Karp X, Ambros V (2009) A feedback circuit involving let-7-family miRNAs and DAF-12 integrates environmental signals and developmental timing in Caenorhabditis elegans. Proceedings of the National Academy of Sciences of the United States of America 106: 18668–18673. 19828440 10.1073/pnas.0908131106 PMC2774035

[88] Zisoulis DG, Kai ZS, Chang RK, Pasquinelli AE (2012) Autoregulation of microRNA biogenesis by let-7 and Argonaute. Nature 486: 541–544. 22722835 10.1038/nature11134 PMC3387326

[89] Bussing I, Slack FJ, Grosshans H (2008) let-7 microRNAs in development, stem cells and cancer. Trends in molecular medicine 14: 400–409. 18674967 10.1016/j.molmed.2008.07.001

[90] Roush SF, Slack FJ (2009) Transcription of the C. elegans let-7 microRNA is temporally regulated by one of its targets, hbl-1. Developmental biology 334: 523–534. 19627983 10.1016/j.ydbio.2009.07.012 PMC2753757

[91] Meissner B, Boll M, Daniel H, Baumeister R (2004) Deletion of the intestinal peptide transporter affects insulin and TOR signaling in Caenorhabditis elegans. The Journal of biological chemistry 279: 36739–36745. 15155758 10.1074/jbc.M403415200

[92] Veljkovic E, Stasiuk S, Skelly PJ, Shoemaker CB, Verrey F (2004) Functional characterization of Caenorhabditis elegans heteromeric amino acid transporters. The Journal of biological chemistry 279: 7655–7662. 14668347 10.1074/jbc.M309528200

[93] Yamagata K, Daitoku H, Takahashi Y, Namiki K, Hisatake K, et al (2008) Arginine methylation of FOXO transcription factors inhibits their phosphorylation by Akt. Molecular cell 32: 221–231. 18951090 10.1016/j.molcel.2008.09.013

[94] Nehrke K (2003) A reduction in intestinal cell pHi due to loss of the Caenorhabditis elegans Na+/H+ exchanger NHX-2 increases life span. The Journal of biological chemistry 278: 44657–44666. 12939266 10.1074/jbc.M307351200

[95] Spanier B, Lasch K, Marsch S, Benner J, Liao W, et al (2009) How the intestinal peptide transporter PEPT-1 contributes to an obesity phenotype in Caenorhabditits elegans. PLoS ONE 4: e6279 doi:10.1371/journal.pone.0006279. 19621081 10.1371/journal.pone.0006279 PMC2708923

[96] Esquela-Kerscher A, Johnson SM, Bai L, Saito K, Partridge J, et al (2005) Post-embryonic expression of C. elegans microRNAs belonging to the lin-4 and let-7 families in the hypodermis and the reproductive system. Developmental dynamics : an official publication of the American Association of Anatomists 234: 868–877. 16217741 10.1002/dvdy.20572 PMC2572564

[97] Johnson SM, Lin SY, Slack FJ (2003) The time of appearance of the C. elegans let-7 microRNA is transcriptionally controlled utilizing a temporal regulatory element in its promoter. Developmental biology 259: 364–379. 12871707 10.1016/s0012-1606(03)00202-1

[98] Martinez NJ, Ow MC, Reece-Hoyes JS, Barrasa MI, Ambros VR, et al (2008) Genome-scale spatiotemporal analysis of Caenorhabditis elegans microRNA promoter activity. Genome research 18: 2005–2015. 18981266 10.1101/gr.083055.108 PMC2593583

[99] Strahl BD, Briggs SD, Brame CJ, Caldwell JA, Koh SS, et al (2001) Methylation of histone H4 at arginine 3 occurs in vivo and is mediated by the nuclear receptor coactivator PRMT1. Current biology : CB 11: 996–1000. 11448779 10.1016/s0960-9822(01)00294-9

[100] Wang H, Huang ZQ, Xia L, Feng Q, Erdjument-Bromage H, et al (2001) Methylation of histone H4 at arginine 3 facilitating transcriptional activation by nuclear hormone receptor. Science 293: 853–857. 11387442 10.1126/science.1060781

[101] Takahashi Y, Daitoku H, Hirota K, Tamiya H, Yokoyama A, et al (2011) Asymmetric arginine dimethylation determines life span in C. elegans by regulating forkhead transcription factor DAF-16. Cell metabolism 13: 505–516. 21531333 10.1016/j.cmet.2011.03.017

[102] Brenner S (1974) The genetics of Caenorhabditis elegans. Genetics 77: 71–94. 4366476 10.1093/genetics/77.1.71 PMC1213120

[103] Irizarry RA, Hobbs B, Collin F, Beazer-Barclay YD, Antonellis KJ, et al (2003) Exploration, normalization, and summaries of high density oligonucleotide array probe level data. Biostatistics 4: 249–264. 12925520 10.1093/biostatistics/4.2.249

[104] Huang da W, Sherman BT, Lempicki RA (2009) Systematic and integrative analysis of large gene lists using DAVID bioinformatics resources. Nature protocols 4: 44–57. 19131956 10.1038/nprot.2008.211

[105] Huang da W, Sherman BT, Lempicki RA (2009) Bioinformatics enrichment tools: paths toward the comprehensive functional analysis of large gene lists. Nucleic acids research 37: 1–13. 19033363 10.1093/nar/gkn923 PMC2615629

[106] Yeo GW, Van Nostrand EL, Liang TY (2007) Discovery and analysis of evolutionarily conserved intronic splicing regulatory elements. PLoS Genet 3: e85 doi:10.1371/journal.pgen.0030085. 17530930 10.1371/journal.pgen.0030085 PMC1877881

