# Supplementary material for: Functional Genomic Analysis of the let-7 Regulatory Network in Caenorhabditis elegans
Source: PLoS Genet. 2013 Mar 14;9(3):e1003353. doi: 10.1371/journal.pgen.1003353 (PMC3597506; doi:10.1371/journal.pgen.1003353)
Supplement: Figure S1 — Seam cell fusion proceeds normally in let-7 mutants. Indirect immunofluorescence with the MH27 monoclonal α-AJM-1 antibody labels apical junctions in hypodermal cells, allowing visualization of seam cell fusion. Fused seam cells are seen in WT (A) and let-7(mn112) (B) at the young adult stage by the lack of junctions between cells (white arrowheads), which are apparent in lin-29(n333) worms where seam cell fusion fails (C). No dramatic decrease in fusion was seen in either let-7(mn112) early/mid L4 worms with (let-7(mn112); Ex[let-7(+);myo-2::GFP]) or without (let-7(mn112)) the rescue fragment (D) or in let-7(n2853) compared to WT worms at the adult stage (E). (DOCX) [file pgen.1003353.s001.docx]

**
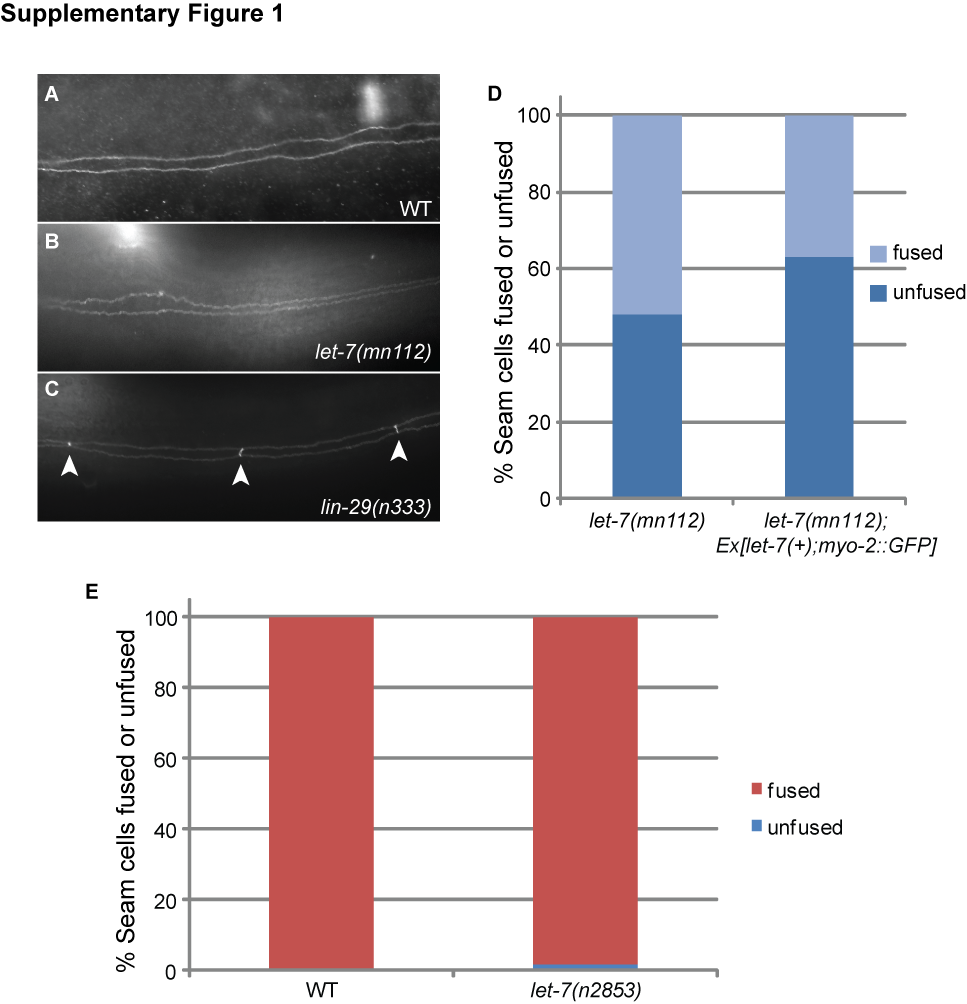
Supplemental Figure 1. Seam cell fusion proceeds normally in *let-7* mutants.** Indirect immunofluorescence with the MH27 monoclonal α-AJM-1 antibody labels apical junctions in hypodermal cells, allowing visualization of seam cell fusion. Fused seam cells are seen in WT (A) and *let-7(mn112)* (B) at the young adult stage by the lack of junctions between cells (white arrowheads), which are apparent in *lin-29(n333)* worms where seam cell fusion fails (C). No dramatic decrease in fusion was seen in either *let-7(mn112)* early/mid L4 worms with (*let-7(mn112); Ex[let-7(+);myo-2::GFP]*) or without (*let-7(mn112)*) the rescue fragment (D) or in *let-7(n2853)* compared to WT worms at the adult stage (E).
