# Supplementary material for: Functional Genomic Analysis of the let-7 Regulatory Network in Caenorhabditis elegans
Source: PLoS Genet. 2013 Mar 14;9(3):e1003353. doi: 10.1371/journal.pgen.1003353 (PMC3597506; doi:10.1371/journal.pgen.1003353)
Supplement: Figure S2 — Conservation of potential let-7 complementary sites (LCSs) in mammalian prmt-1. (A) Genome browser track showing the last exon of PRMT1. Base-wise conservation from PhastCons is shown in green for all species with available alignments on the UCSC Genome Browser. 3′UTR locations complementary to let-7 are drawn as black rectangles. (B) PRMT1::let-7 duplexes predicted by RNAhybrid. Capital letters denote paired bases, lower-case letters indicate unpaired bases, dashes indicate gaps. Minimum free energy of binding is listed on the right side of each duplex. (C) All five LCS positions and their conservation across the available genome alignments is shown. Dots indicate an exact match to the human reference. Vertical orange lines indicate insertions or deletions with the size of gaps listed along the bottom of each alignment. (DOCX) [file pgen.1003353.s002.docx]

**Supplemental Figure 2. Conservation of potential let-7 complementary sites (LCSs) in mammalian prmt-1.** (A) Genome browser track showing the last exon of PRMT1. Base-wise conservation from PhastCons is shown in green for all species with available alignments on the UCSC Genome Browser. 3’UTR locations complementary to let-7 are drawn as black rectangles. (B) PRMT1::let-7 duplexes predicted by RNAhybrid. Capital letters denote paired bases, lower-case letters indicate unpaired bases, dashes indicate gaps. Minimum free energy of binding is listed on the right side of each duplex. (C) All five LCS positions and their conservation across the available genome alignments are shown. Dots indicate an exact match to the human reference. Vertical orange lines indicate insertions or deletions with the size of gaps listed along the bottom of each alignment.

**
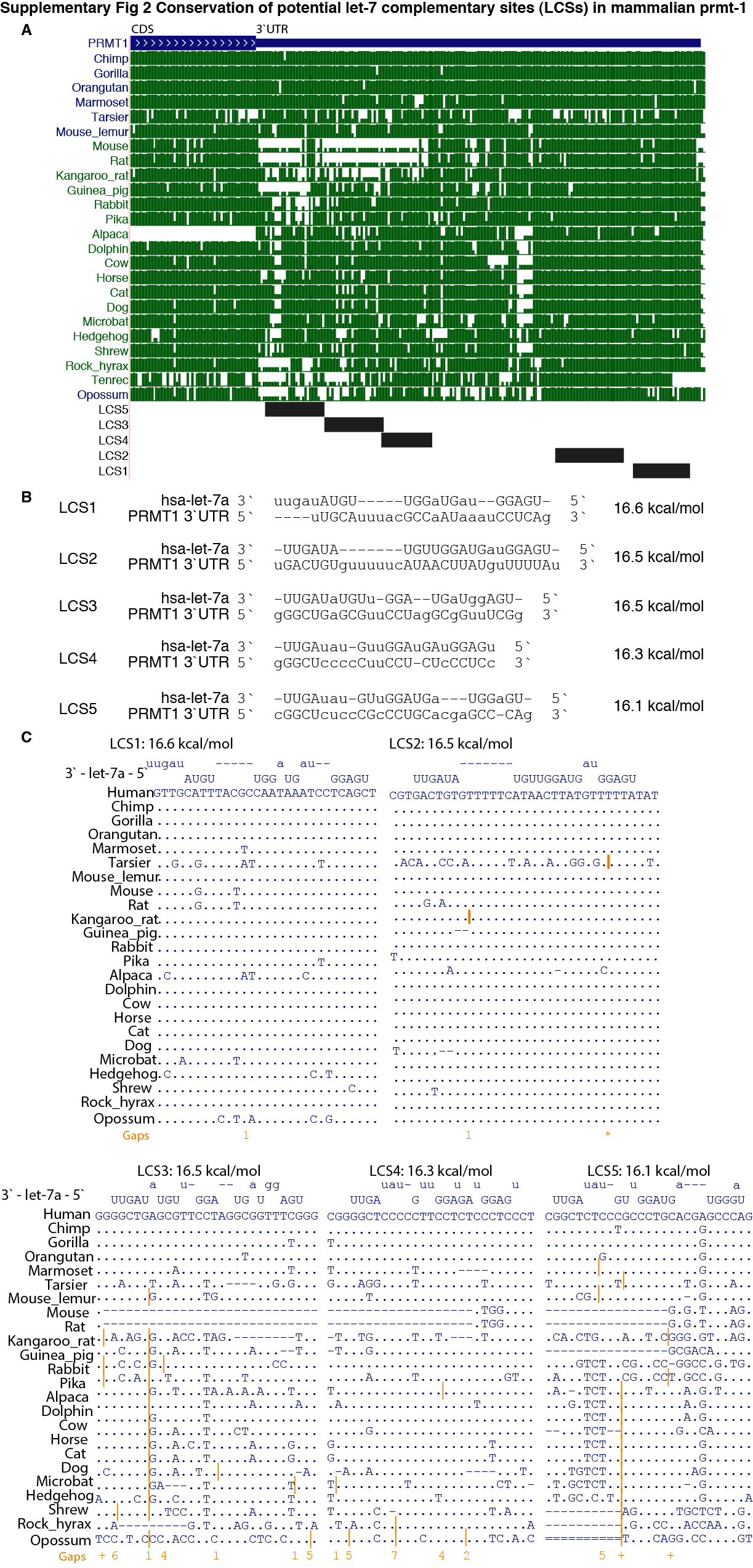
**
